# Fully Textured Monolithic Sb_2_S_3_/Silicon Tandem for Unbiased and Stable Solar‐Driven Water Splitting Paired with Iodide Oxidation Reaction

**DOI:** 10.1002/advs.75798

**Published:** 2026-05-22

**Authors:** Jihong Min, Irene Dei Tos, Sepideh Rahimisheikh, Beatriz de la Fuente, Devika Rajagopal, Jan D'Haen, David Cornil, Tom Hauffman, Tom Aernouts, David Beljonne, Joke Hadermann, Byungha Shin, Bart Vermang, Sudhanshu Shukla

**Affiliations:** ^1^ Department of Materials Science and Engineering Korea Advanced Institute of Science and Technology Daejeon Republic of Korea; ^2^ imec IUMAT Genk Belgium; ^3^ UHasselt Institute for Materials Research (IUMAT) Hasselt Belgium; ^4^ EnergyVille Genk Belgium; ^5^ Electron Microscopy for Materials Science (EMAT) University of Antwerp Antwerpen Belgium; ^6^ Research Group Sustainable Materials Engineering (SUME) Lab of Electrochemical and Surface Engineering (SURF) Vrije Universiteit Brussel Brussels Belgium; ^7^ UHasselt Institute for Materials Research (IUMAT) Analytical & Microscopical Services (AMS) Hasselt Belgium; ^8^ imec IUMAT Diepenbeek Belgium; ^9^ Laboratory for Chemistry of Novel Materials University of Mons (UMONS) Mons Belgium

## Abstract

Solar‐driven photoelectrochemical (PEC) production of chemical fuels such as hydrogen is a viable solution to address climate neutrality objectives. Development of a monolithic tandem PEC device consisting of ideal bandgap absorbers is of paramount importance to realize efficient artificial photosynthesis systems. Herein, we report monolithic integration of Sb_2_S_3_ on textured silicon to realize a completely inorganic and fully vacuum processed multilayer PEC device with Ag/Indium Tin Oxide (ITO)/Heterojunction with Intrinsic Thin layer (HIT) Si/ITO/Au/Sb_2_S_3_/NiO_x_ architecture. Photoelectron spectroscopy and computational analysis show a staggered band alignment between Si and Sb_2_S_3_, emulating Z‐scheme charge transfer mechanism. We demonstrate a high performing and stable Sb_2_S_3_‐Si monolithic tandem for PEC hydrogen evolution reaction (HER) coupled to iodide oxidation reaction (IOR). Under AM 1.5G illumination, the Sb_2_S_3_‐Si monolithic tandem device achieves unassisted photocurrent density of 4.38 mA cm^−^^2^ with faradaic efficiency of 97% for hydrogen, while maintaining ∼90% of its initial performance after 10 h of continuous operation. These results set a new benchmark for all inorganic monolithic tandems for efficient and sustainable solar‐to‐chemical conversion. This work unlocks the pathway for artificial photosynthesis systems comprising ideal bandgap photo absorbers.

## Graphical abstract

Monolithically integrated Sb_2_S_3_ on industry‐compatible textured silicon for tandem photoelectrochemical hydrogen evolution coupled with iodide oxidation.

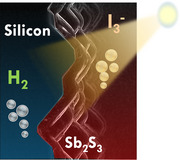

## 1 Introduction

Fossil fuel‐dependent growth has led to an unprecedented rise in the atmospheric CO_2_ levels. This has triggered a climate emergency, which has emerged as one of the biggest global challenges of our time. Therefore, a paradigm shift to renewable energy is urgently required to defossilize the economy and ensure carbon‐neutral and sustainable growth [1]. Photoelectrochemical (PEC) water splitting is a highly promising technology for achieving a carbon‐zero energy system, as it directly converts solar energy into chemical fuels by utilizing photoexcited electrons and holes generated in illuminated semiconductor materials [2]. The thermodynamic energy required to split a water molecule is 1.23 eV. However, the cumulative energy requirements exceed beyond ∼1.8 eV mainly due to overpotential losses associated with the poor kinetics of the oxygen evolution reaction (OER), which involves multi‐step proton‐coupled electron transfer processes [3]. Recently, PEC‐driven iodide oxidation reaction (IOR) has gained significant attention due to its faster kinetics than OER with almost negligible overpotential [4, 5]. In particular, iodine (I_2_) produced from IOR is a value‐added chemical with significant economic advantages (market cost of 59 $/kg) [6] compared to oxygen, and it is widely utilized in nuclear medicine applications, such as thyroid diagnostics and medical imaging, as well as in energy storage systems like Zn–iodine batteries [7, 8, 9].

**FIGURE 1:**
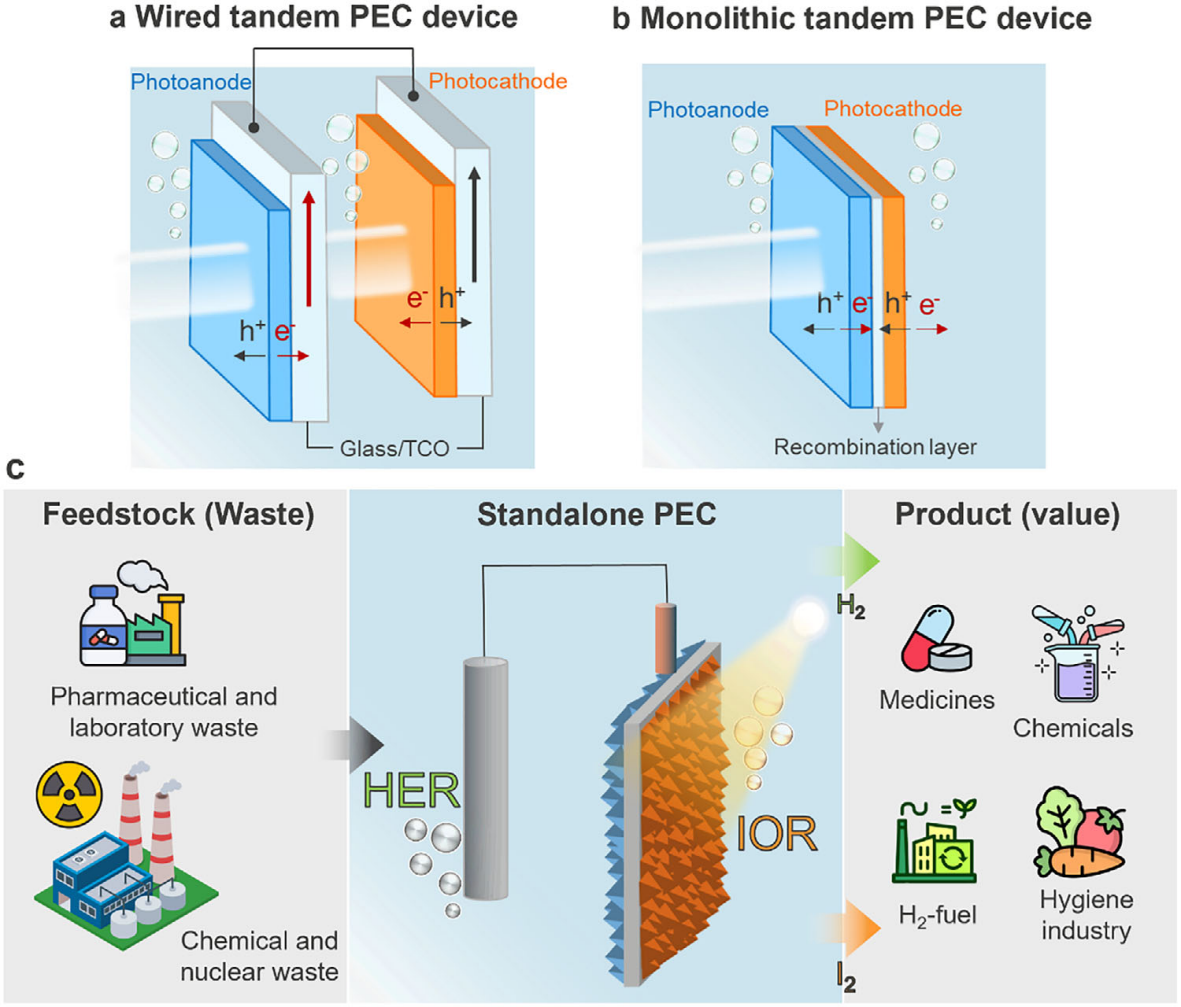
Schematic illustration of two tandem configurations; a) Wired tandem PEC device, where significant ohmic losses occur due to lateral charge transport through TCO layers, and b) Monolithic tandem PEC device representing ohmic‐loss‐free configuration, where photogenerated charges cover shorter distances before they recombine or are utilized in a catalytic reaction. c) Schematic diagram of the role of stand‐alone PEC device for hydrogen generation coupled to alternative value‐added iodide oxidation reaction.

For high performing PEC cell coupling HER and IOR, stacking light absorbers of appropriate energy bandgap in a tandem configuration is needed to maximize the capture of incident solar photons and simultaneously generating high enough photovoltage for stand‐alone operation [10, 11]. The two main tandem configurations are (a) wired or mechanically stacked tandem and (b) monolithic tandem, as shown in Figure 1a,b (Text S2). The latter configuration (b) resolves efficiency losses typically encountered when scaling up PEC devices and considerably reduces cost burdens associated with expensive and scarce indium or silver containing transparent conductive oxide (TCO) layers. Thus, a monolithic tandem could substantially enhance the techno‐economic characteristics and simplicity of solar‐driven hydrogen production systems when coupled to alternative IOR, as shown in Figure 1c. However, monolithic integration poses stringent requirements on the materials, such as suitable bandgaps, efficient charge transport, and processing compatibility. Moreover, absorber instability in aqueous electrolytes presents significant challenge. [12, 13] Therefore, meeting this requirement necessitates developing robust surface passivation or protection layers, involving complex and additional fabrication steps.

Detailed balance analysis reveals an optimum combination of ∼1.7 eV (top absorber) and ∼1.1 eV (bottom absorber) bandgap to maximize the solar spectrum utilization and achieve a theoretical solar‐to‐hydrogen (STH) efficiency of >25% under AM 1.5G 1‐Sun illumination. [14] For the bottom cell in tandem PEC devices, the choice of silicon is straight‐forward by virtue of its ideal bandgap and the ongoing success of the c‐Si industry in terms of improved performance and its declining cost in photovoltaic (PV) devices [15]. A fully (double‐sided) textured silicon is even more attractive due to its light‐trapping benefits and higher surface roughness, both being ideal for photocatalytic applications [16]. However, attempts to deposit top absorbers directly on silicon for monolithic tandem devices have faced challenges related to the high processing temperature and the suboptimal bandgaps of commonly used wide bandgap absorbers for photoelectrochemistry, such as metal oxides. Also, the deposition methods are often incompatible with the rough surfaces of textured silicon. These stringent requirements preclude the monolithic integration of top absorbers on silicon, limiting the most current approaches to wire‐connected mechanically stacked tandems. As a rare example of successful monolithic integration, a silicon‐BiVO_4_ tandem has been reported by Jung et al. using a flat TOPCon silicon [17]. In realizing an ideal monolithic tandem PEC device, the main challenge lies in developing an efficient and stable top absorber, along with a tailored deposition that enables monolithic integration onto textured silicon. This process should preserve the optoelectronic quality and material integrity of silicon while ensuring conformal coverage and atomically smooth interfaces. Meeting all these criteria simultaneously is exceedingly difficult, and the practical realization of this concept requires overcoming numerous technical challenges.

Recently, binary quasi‐1D (Q1D) orthorhombic antimony trisulfide (Sb_2_S_3_) has garnered considerable attention in PV and PEC devices due to its desirable physical and optoelectronic properties, such as high optical absorption coefficient (∼10^5^ cm^−1^), and long hole diffusion lengths, and high thermal and environmental stability [18, 19]. In particular, its bandgap of 1.70 eV is nearly ideal for use as the top cell in tandem devices when paired with silicon. Furthermore, relatively lower temperature processability (crystallization at ∼ 300°C) compared to other inorganic semiconductors makes it ideal for monolithic integration with silicon. Studies indicate that Sb_2_S_3_ exhibits slightly n‐type character, making it conducive to driving oxidation reactions on Sb_2_S_3_ photoanodes, while concomitant reduction reactions can occur on silicon photocathodes [20]. A few studies have explored archetypical OER and IOR anodic reactions on Sb_2_S_3_ photoanodes and coupled it to the hydrogen evolution reaction (HER) on silicon or perovskite photocathodes [4, 21, 22]. While these Sb_2_S_3_‐Si tandem architectures are demonstrated to work, the reported PEC devices mainly relied on a mechanically stacked tandem configuration, with no demonstrated success in achieving monolithic tandem integration of Sb_2_S_3_ on silicon.

Despite vital necessities for an ideal tandem device, the Q1D crystal structure of Sb_2_S_3_ poses charge transport limitations. The [Sb_4_S_6_] units are linked into infinite covalently bonded [Sb_4_S_6_]_n_ ribbons along the *c*‐axis. Neighboring [Sb_4_S_6_]_n_ ribbons are linked through weak van der Waals (vdW) interactions to form crumpled sheets perpendicular to the *b*‐axis [23]. This intrinsically reduced dimensionality leads to anisotropy in its electronic properties, with two orders of magnitude higher charge carrier mobility and conductivity along the ribbon versus perpendicular to the sheets of ribbons [18, 24, 25]. Therefore, [hk1] oriented films (where the sheets lie diagonally to the substrate) are preferred to achieve high‐performing devices. However, the low energy vdW (hk0) crystal surfaces tend to grow parallel to the substrate, which hinders efficient vertical charge transport and results in low carrier lifetimes [26, 27]. In order to grow [hk1]‐oriented Sb_2_S_3_ films, doping and substrate engineering strategies have been pursued mainly in hydrothermally grown films. For instance, doping with Ag, Se, and Cl [28, 29], or modified growth on specific substrates like CdS, TiO_2_ [24, 30, 31, 32]. In contrast, there has been limited success in achieving oriented growth in vacuum deposition approach [33], which is desirable for scaling and mass‐production.

In this work, we have taken advantage of the low melting temperature and high vapour pressure of Sb_2_S_3_ to build a conformal Sb_2_S_3_‐Si tandem PEC device via physical vapor deposition with enhanced charge separation and light management. A high‐crystalline quality sub‐micron‐thick Sb_2_S_3_ photoanode was successfully grown conformally on a textured heterojunction with intrinsic thin layer (HIT) silicon stack and found that the film consists of large monolithic grains. Furthermore, a hole transport layer (HTL) of NiO_x_ characterized for suitable energy band alignment with Sb_2_S_3_ was applied to facilitate the hole transfer at the surface. The resulting monolithic tandem photoelectrode was tested for photoelectrochemical hydrogen production coupled with IOR. The charge transport and stability of the top Sb_2_S_3_ layer improved significantly by introducing a dual‐layer passivation comprising of thin and transparent protective titanium (Ti) layer on top of HTL layer (NiO_x_). Finally, the monolithically integrated Sb_2_S_3_–Si tandem photoelectrode, connected to a Pt cathodic catalyst via external wiring, successfully demonstrated unbiased hydrogen and iodine co‐production. Under AM 1.5G (1 sun) illumination, the tandem device delivered an excellent photocurrent density of 4.38 mA/cm^2^ and retained 90% of its initial photocurrent density after 10 h of unassisted operation. Taking this into consideration, we demonstrate the successful monolithic integration of Sb_2_S_3_ and Si absorbers toward tandem PEC device for highly efficient and stable hydrogen generation paired with iodide waste upcycling in strong acid (Figure 1b).

## 2 Results and Discussions

### 2.1 Conformal Deposition of Sb_2_S_3_ on Textured Silicon

**FIGURE 2:**
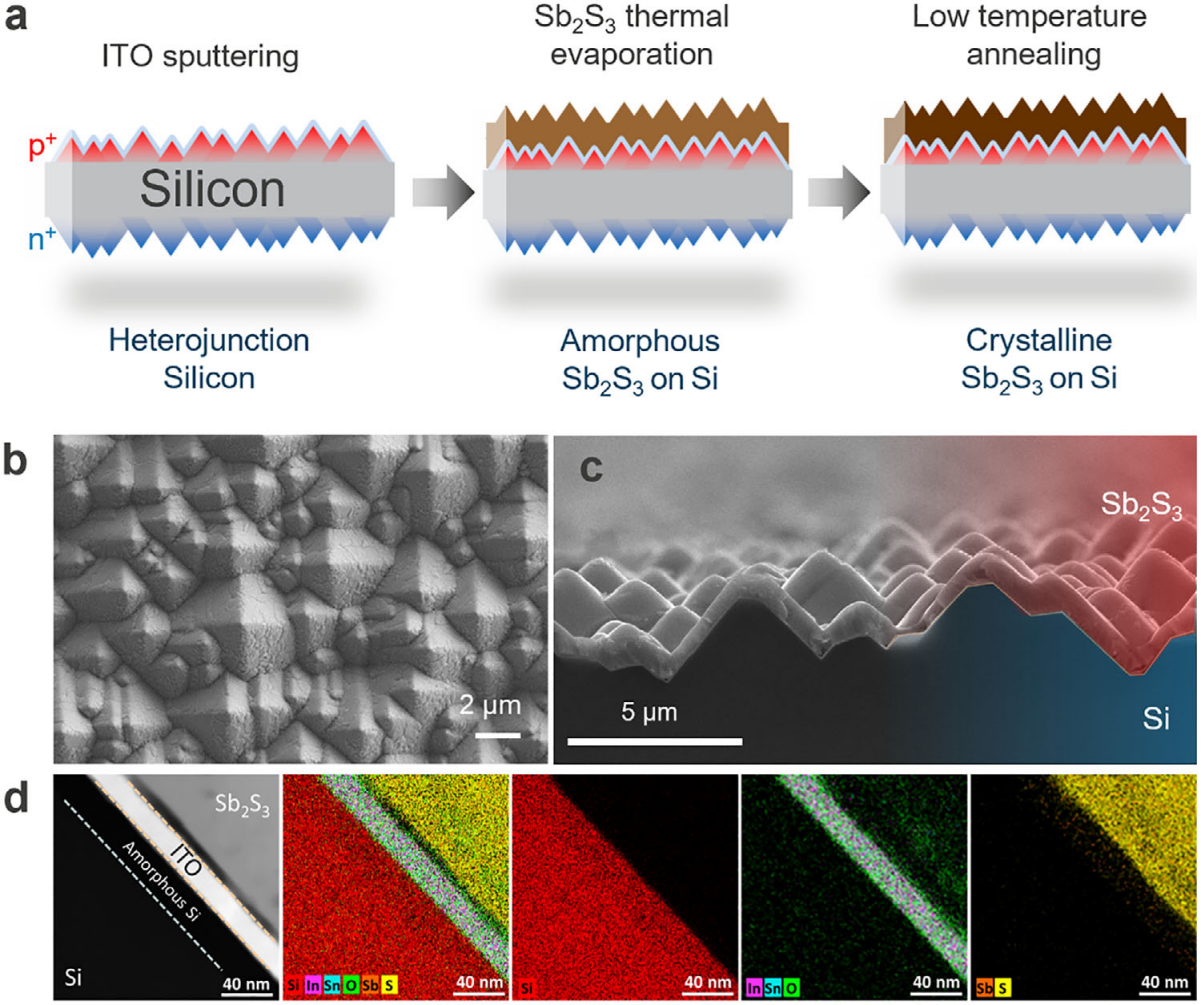
Fabrication process and microstructure of Sb_2_S_3_–Si monolithic tandem stack. a) Schematics of the deposition process of Sb_2_S_3_ over textured silicon in n‐i‐p configuration. b) Top‐view, and c) cross‐section SEM of the textured silicon surface coated with Sb_2_S_3_. Microstructural characterization and properties of Sb_2_S_3_ film deposited on textured silicon. d) High‐angle annular dark‐field (HAADF) image and corresponding STEM‐EDX maps showing the elemental distribution of all elements, and of the elements targeted for the different layers separately.

Figure 2a schematically illustrates the deposition of the Sb_2_S_3_ layer on a fully textured heterojunction (111) silicon bottom sub‐cell in n‐i‐p tandem configuration. The front surface of the silicon cell was capped by a thin passivating indium tin oxide (ITO) layer, which also functions as a recombination junction in a tandem device (Figure S1).

A Sb_2_S_3_ thin film was deposited in a 2‐step sequential process; first, evaporating the Sb_2_S_3_ precursor powder on the substrate (kept at room temperature) to form the precursor amorphous film, followed by sulfurization in a tube furnace at mild temperature of 300°C to complete the crystallization. More information can be found in the experimental details, Text S1. The thermal evaporation process provides good control over the thickness of the Sb_2_S_3_ and yields conformal and full coverage over the silicon pyramids, enabling precise nanoscale interfaces. As evident from top‐view and cross‐section scanning electron microscope (SEM) images in Figure 2b,c, respectively, the deposition process yields uniform and dense Sb_2_S_3_ film of thickness of approximately 0.5 𝜇m over the silicon pyramids. Strikingly, the microstructure and the large grain features on textured silicon substrate are similar to those obtained on planar indium tin oxide (ITO) or fluorine‐doped tin oxide (FTO)‐coated soda‐lime glass substrates, demonstrating the robustness of the deposition process (Figure S2). Notably, layer thickness of ∼500 nm is judiciously fixed in the current study to ensure adequate light absorption.

We examined the elemental distribution and conformality at the nanoscale interfaces by means of cross‐section high‐angle annular dark‐field scanning transmission electron microscopy (HAADF‐STEM) and corresponding energy dispersive x‐ray spectroscopy (EDS). The analysis reveals a precisely defined and smooth Si/ITO/Sb_2_S_3_ interface with excellent coverage, showing no evidence of undesirable interdiffusion of Sb/S in silicon, Figure 2d (see also Figure S3). The darker region between ITO and Sb_2_S_3_ indicates an area with less density. TEM analysis also reveals that the doped amorphous region in the silicon heterojunction remains intact after the Sb_2_S_3_ deposition process, visible as a slightly darker band in the Si layer in Figure 2d (and better visible on Figure S4).

**FIGURE 3:**
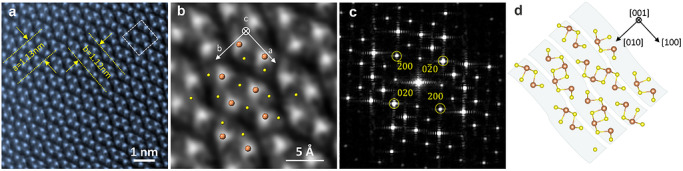
a) Atomic‐resolution image of the Sb_2_S_3_ part in the stack projected along the [001] direction. b) Higher magnification experimental image overlayed with the reference model of the Sb_2_S_3_ structure [34], sulfur atomic columns in yellow (low contrast sites) and Sb atomic columns in orange (brighter contrast sites), c) corresponding FFT pattern of orthorhombic Sb_2_S_3_, and d) schematic illustration of the crystal structure. The [Sb_4_S_6_]_n_ sheets are indicated with light green bands.

Figure 3a shows an atomic resolution image of the Sb_2_S_3_ lattice viewed along the [001] projection. This high‐resolution image was taken from another sample than the overview image, cut along a different orientation. The unit cell is indicated by a dotted square in Figure 3a, with a close‐up in Figure 3b. Based on the FFT (Figure 3c), the cell parameters are a = 11.64(9) Å and b = 11.7(4) Å, while literature cell parameters for Sb_2_S_3_ are a = 11.25(2) Å, b = 11.33(2) Å, and c = 3.83(1) Å. No statement can be made about the length of the *c*‐axis in the current sample, as the orientation of the cut did not allow to reach a diffraction pattern containing the c*‐axis. HAADF‐STEM images and their FFTs can be affected by lens aberrations and voltage instabilities, which could explain the slightly higher cell parameters. The reflection conditions visible on the FFT agree with the *Pbnm* space group from literature (visible for [001] only: *hk0* = no conditions, *h00: h = 2n* and *k00: k = 2n*), as shown in the model in Figure 3d.

### 2.2 Crystallinity and Preferred Orientation

**FIGURE 4:**
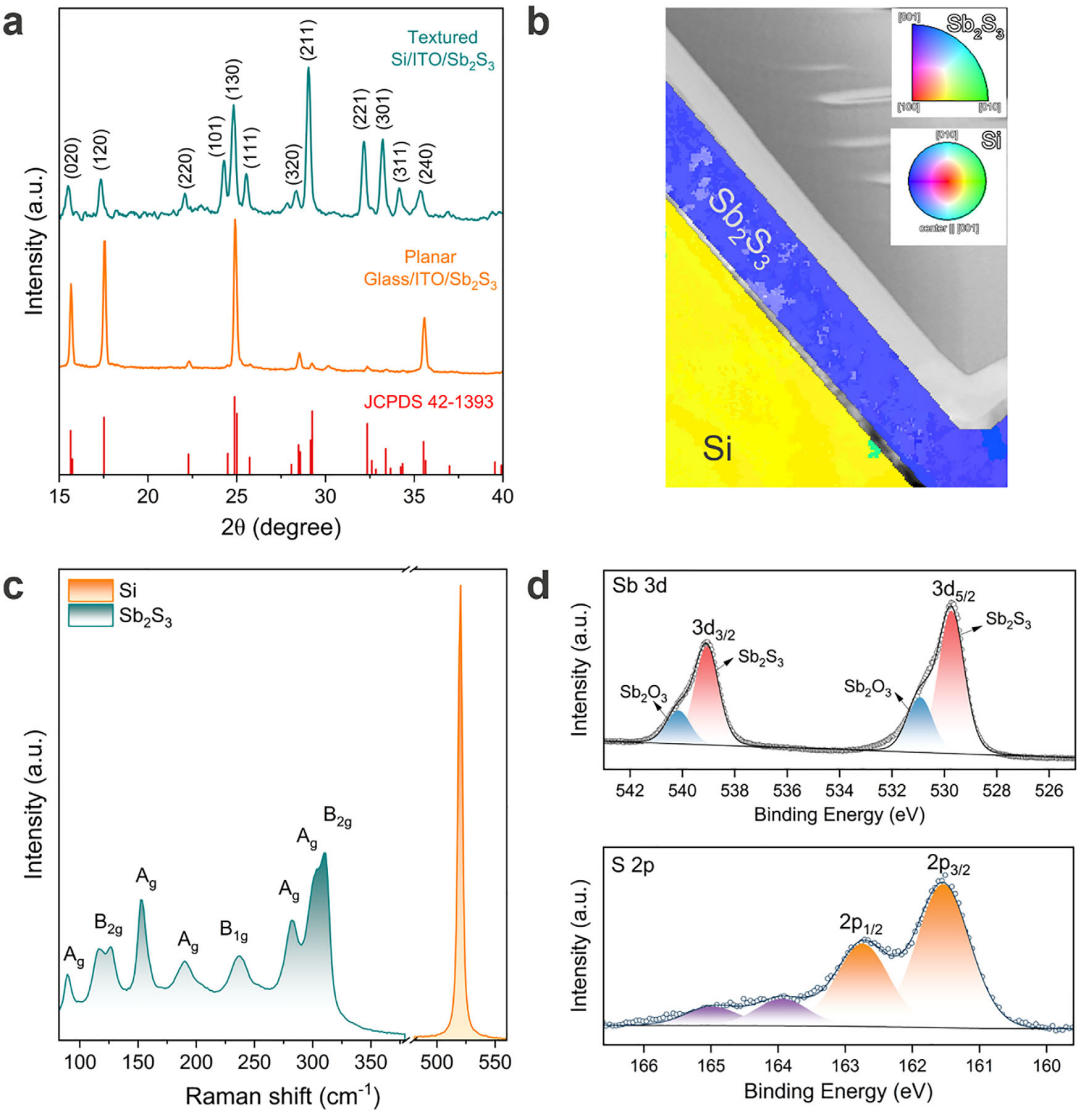
Structural characterization and crystal orientation analysis of Sb_2_S_3_ film deposited on textured silicon. a) X‐ray diffraction (XRD), b) crystal orientation map based on electron diffraction data. c) Raman spectra, and d) high‐resolution XPS spectra of Sb 3d and S 2p core levels of the Sb_2_S_3_ thin film, fitted with multiple Voigt peaks (shaded areas), on (111) textured silicon substrate.

X‐ray diffraction (XRD) and Raman spectroscopy were performed to analyze crystallinity and phase. Figure 4a shows the XRD diffractogram of the Sb_2_S_3_ film on the textured silicon substrate. All the diffraction peaks can be indexed to orthorhombic stibnite Sb_2_S_3_ phase with a space group *Pbnm* (JCPS #42‐1393). The high crystalline quality is evidenced by the sharp intensity of the peaks. The reflections corresponding to (hk1) crystal planes i.e. (101), (111), (211), (221), (301), and (311) are much more pronounced in thin films grown on textured silicon substrate than in those grown on planar ITO/glass and ITO/polished‐silicon substrates, as evident from the XRD in Figure 4a and Figure S5, where the (hk0) reflections are much stronger.

This implies that the 1D ribbons of the Sb_2_S_3_ film on textured silicon substrate tend to grow perpendicular or tilted relative to the substrate, which is beneficial for the carrier collection and also reduces carrier recombination due to absence of dangling bonds at the grain boundaries (GBs) parallel to the c‐axis [18]. Most of the reports pertaining to oriented growth are based on the hydrothermal deposition method and specific substrates such as CdS or TiO_2_ [24, 33, 35]. For vapor deposited Sb_2_S_3_, a study by Zhang et al. demonstrates the control over the orientation on CdS coated substrates using vapor transport deposition (VTD) process, although requiring a high substrate temperature of > 380°C [33]. The study attributed the dynamic evaporation and re‐evaporation mechanism to explain the orientation change. We performed additional microscopic measurements at the interface to gain deeper insights into the origin of this apparent orientation change in XRD measurements. Figure 4b shows the orientation map of the full area in the image. The blue shade in the coating region indicates that the crystals are close to the [001]‐zone axis, and since the shade is almost flat, the grain orientation is homogeneous throughout the analyzed Sb_2_S_3_ film. Si substrate also shows a homogeneous orientation of the single‐crystal silicon. Neglecting the flat tops of the pyramids with the <100> orientation, the above analysis implies that the interface of the substrate is parallel to the c‐axis (hk0 planes) of the film, as shown in Figure 4b (see also Figure S6). Interestingly, no grain boundaries (GBs) appear in the orientation map, indicating the monolithic nature of Sb_2_S_3_ grains. More diffraction peaks corresponding to different (hk0) planes in the XRD simply indicate that these (hk0) planes are rotated over different facets of the pyramid. Thus, the XRD results comply with the STEM analysis. Thus, the increase in (hk1) reflections in XRD can be explained by geometrical consideration of the interface. We conclude that the film does not reorient on textured surface, but the reflections in XRD change due to the angle of the textured facets of the Si pyramids.

Complementing the XRD analysis, we resorted to Raman spectroscopy to assess the structure and chemical bonds. Despite the seemingly simple binary compound, the Raman spectra are complex due to different coordination environments of Sb‐ and S‐ sites and hence different bonds. Group theory predicts 30 Raman‐active modes for Sb_2_S_3_, i.e., Γ  =  10*A_g_* + 5*B*_1*g*_ + 10*B*_2*g*_ + 5*B*_3*g*_ [36]. Several phonon modes were clearly resolved in the spectra, indicating good crystalline quality of the film, as displayed in Figure 4c. Raman peaks at 89, 116, 152, 190, 236, 282, 303, and 309 cm^−1^ are assigned to A_g_, B_2g_, A_g_, A_g_, B_1g_, A_g_, A_g_, and B_2g_ phonon modes. The characteristic modes at 190 and 236 cm^−1^ correspond to the S‐Sb‐S anti‐symmetric and symmetric bending vibrations, respectively. The asymmetric and symmetric stretching vibrations of the Sb─S bond were observed at 282 and 303 cm^−1^ respectively [37]. Additionally, an intense peak at 520 cm^−1^ is ascribed to the characteristic phonon scattering from silicon substrate [38]. X‐ray photoelectron spectroscopy (XPS) measurements were carried out to gain chemical insights about the surface of Sb_2_S_3_. Figure 4d shows high‐resolution Sb 3d and S 2p core level spectra (full survey scan in Figure S7). Peaks at the binding energy at 593.0 and 529.7 eV could be attributed to Sb 3d_3/2_ and Sb 3d_5/2_ respectively, consistent with the Sb^3+^ valency in Sb_2_S_3_. The additional shoulder peak at 540.1 and 530.9 eV corresponds to Sb 3d_3/2_ and Sb 3d_5/2_ of Sb_2_O_3_ [39]. It could stem from the surface oxidation caused by air exposure. S 2p spectra shows two sulfur species, each fitted as a doublet with spin–orbit splitting of 1.2 eV. The binding energy at 161.5 and 162.7 eV are ascribed to S 2p_3/2_ and S 2p_1/2,_ respectively, consistent with the disulfide species (S^2−^) in Sb_2_S_3_ [40]. Additional small peak (purple doublet) at 163.9 and 165.1 eV is most likely due to oxidized sulfur species [41]. The findings underscore the purity and excellence of the deposited films on textured silicon substrate.

### 2.3 Tandem Stack Design and Energetics for Photoelectrochemical Reaction

**FIGURE 5:**
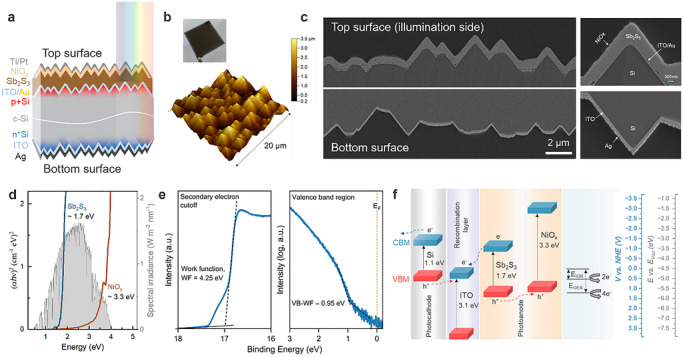
Tandem PEC device design, microstructure, optical properties and energy band alignment analysis. a) Schematic view of the monolithic Pt/Ti/Sb_2_S_3_/Au/ITO/Si/ITO/Ag (top to bottom) tandem PEC device built using a double side textured silicon heterojunction stack. b) 3D representation of front surface morphology scanned from AFM. The inset shows the photograph of a complete tandem cell. c) cross‐section SEM image of front (top) and back (bottom) surface of the full tandem stack along with the magnified view of pyramidal 30 nm NiO_x_/ 500 nm Sb_2_S_3_/ 10 nm Au/ 25 nm ITO layers at the front and 100 nm ITO/ 100 nm Ag layers at the back. d) Tauc plot of NiO_x_ and Sb_2_S_3_ showing minimum loss of photons from top NiO_x_ layer. The AM 1.5G spectrum is included as a reference. e) Ultraviolet (He I, excitation energy of 21.1 eV) photoelectron spectra (UPS) of the Sb_2_S_3_ film showing the work function calculated from secondary electron cut‐off and valence band offset relative to Fermi level calculated from valence band region. f) Energy band alignment of Si/ITO/Sb_2_S_3_/NiO_x_ and relative position of the OER and IOR redox potentials.

To construct a full tandem device consisting of a Sb_2_S_3_ photoanode and a silicon photocathode, we applied a sputter‐deposited ultrathin (∼30 nm) NiO_x_ hole transporting layer (HTL) on top of the Sb_2_S_3_. The NiO_x_ layer also serves as surface protection to the absorber. An ultrathin layer of Au (∼10 nm) was inserted to enhance the majority carrier recombination at the ITO recombination junction while ensuring adequate light penetration. Moreover, it has been reported that Au forms an ohmic contact to Sb_2_S_3_ [42]. On the silicon back side, an Ag back contact was used to collect photogenerated electrons for HER. A schematic representation of the full tandem stack is shown in Figure 5a. Vacuum processing enables scalable and reproducible fabrication on large‐area textured silicon substrates. The vapor flux distribution reaching the substrate can be made spatially uniform over extended areas, enabling accurate nanometer‐scale thickness control and conformal coating even on large‐area substrates [43, 44]. Owing to these advantages of vacuum deposition, the present stack exhibits homogeneous film formation over a large area of 3 × 3 cm^2^ while retaining the faceted texture of the micrometer‐sized Si pyramids, as evident from the atomic force microscopy (AFM) image shown in Figure 5b.

The cross‐section SEM images of the top and bottom interfaces of the tandem device after focused ion beam (FIB) milling from a relatively large scanning area, showing clearly the respective transport layers (Figure 5c). The detailed view of individual layers in the tandem stack can be found in Figure S8. The large bandgap of NiO_x_ (∼3.3 eV) top layer ensures sufficient light transmission to the underlying Sb_2_S_3_ and Si layers (Figure 5d). A suitable energy band alignment is essential for effective charge separation in a multilayered device. We conducted ultraviolet photoelectron spectroscopy (UPS) to probe the energy band positions of Sb_2_S_3_ and NiO_x_ and combined the bandgap information derived from Ultraviolet‐visible (UV–vis) spectroscopy to construct the full energy band alignment. The linear extrapolation of the secondary energy cut‐off provides the work function (*W_F_*) value of 4.25 eV (Figure 5e). Full UPS scan is provided in Figure S9. From the logarithmic extrapolation of the leading edge, the valence band offset relative to the Fermi level (*E_VBM_* − *E_F_*) was determined to be 0.95 eV (Figure 5f). Such a high energetic difference between VBM and the Fermi level indicates weakly n‐type nature of the Sb_2_S_3_. While the relatively deep Fermi energy (i.e., nearly intrinsic) allows the Sb_2_S_3_ to function as both a photocathode and a photoanode, and its slightly n‐type conductivity makes it more suitable for photoanodic applications. By taking into account the measured bandgap values of all the layers in the stack and the energy band positions of silicon from literature [45, 46], the energy band diagram of the Si/ITO/Sb_2_S_3_/NiO_x_ is outlined in Figure 5f, illustrating favorable charge transfer. Given the staggered band alignment between Si and Sb_2_S_3_, a Z‐scheme charge transfer mechanism is proposed, where the photogenerated electrons at the CBM of Sb_2_S_3_ and photogenerated holes at the VBM of Si readily recombine at the ITO interface (see also Figure S10). Computational analysis shows strong wavefunction hybridization at the Sb_2_S_3_/NiO_x_ interface and favorable energy level alignment for hole extraction, The conduction band maximum (CBM) of Sb_2_S_3_ is located 0.5 eV deep into the nickel oxide bandgap, overall resulting in a type II heterojunction (see Figure S11), which provides driving force for the holes transfer and prevents surface recombination due to electron accumulation. Subsequently, the retained photogenerated holes at the VBM of the Sb_2_S_3_ are collected by NiO_x_ to drive oxidation reaction. The Sb_2_S_3_/NiO_x_ surface is principally suited for typical oxidation reactions as the valence band position of Sb_2_S_3_ and NiO_x_ are well aligned with the thermodynamic redox potentials of OER and IOR reaction, with latter requiring less energy input and two electron transfer processes compared to four electron transfer OER process. At the ITO/Sb_2_S_3_ interface, The VBM of the indium oxide cluster is located 0.7 eV below the VBM of Sb_2_S_3_, while its CBM is 0.3 eV deeper than that of Sb_2_S_3_ CBM, again corresponding to a type II heterojunction and providing the necessary driving force for electron extraction by the indium oxide (see Figure S11). Thus, the retained photogenerated electrons at the CBM of Si are collected at the ITO/Ag interface to drive reduction reaction at the counter electrode. All together, these results support the proposed Z‐scheme operation for the inorganic tandem PEC system.

### 2.4 Photoelectrochemical Performance of the Tandem PEC for IOR

It has been previously reported that a Ti‐based |protection layer applied to tunnel oxide passivated contact (TOPCon) silicon photoanodes effectively stabilize Si photoelectrodes in acidic electrolytes while maintaining high optical transparency [47]. Inspired by this strategy, we introduced a 10 nm Ti protection layer deposited by electron‐beam (e‐beam) evaporation onto the NiO_x_ layer to enhance the long‐term stability of our PEC device. Additionally, a thin Pt catalyst layer (3 nm) was deposited onto the Ti protection layer to further improve the kinetics of the IOR. Although Pt is a noble metal, it is used here only as an ultrathin functional layer, such that its direct material‐cost contribution remains limited relative to that of the absorber stack [48]. Moreover, several viable strategies may be considered in future device designs to reduce or replace noble‐metal catalysts. For instance, earth‐abundant Mo‐based catalysts have shown promising IOR activity and stability in related systems, indicating their potential as alternative anodic catalysts [49].

HAADF and STEM‐EDX analyses confirmed that both the Ti protection and Pt catalyst layers were conformally and uniformly deposited (Figure S12). Subsequent chronoamperometry (CA) tests under unbiased conditions (Figures S13 and S14) clearly demonstrate that the stability of the tandem device significantly improved after applying the Ti protection layer compared to the unprotected device. The primary role of the Ti layer is chemical protection, which enables stable anodic operation and thereby allows reliable electrochemical characterization, rather than serving as an intentional strategy to enhance intrinsic charge transport. Notably, the interfacial charge transfer between the NiO_x_/Ti is most plausibly enabled by defect‐rich TiO_x_ (and associated recombination‐assisted pathways) [47, 50], rather than by an oversimplified band‐edge alignment model. The Ti/Pt top layer introduces non‐negligible parasitic optical losses, offering approximately 60%–70% transmittance across the relevant visible to near IR window, Figure S15a. Although the optical penalty of the Ti/Pt protection layer is partially mitigated by the textured silicon substrate [51, 52], non‐negligible optical loss through the protection layer still persists and reduces the theoretical photocurrent. Assuming near‐ideal integration between the Sb_2_S_3_ top absorber and the Si bottom absorber, the maximum operating photocurrent density of the tandem stack would be limited to 14.25 mA cm^−2^ by the optical losses induced by the Ti/Pt protection layer. Future work should therefore explore alternative transparent protection layer strategy, such as coating with TiO_2_ [53], TaO_x_ [54], and polymeric conjugated polycarbazole frameworks [21], to minimize optical attenuation while maintaining chemical robustness. This represents a clear next step toward maximizing photon utilization in this device platform.

**FIGURE 6:**
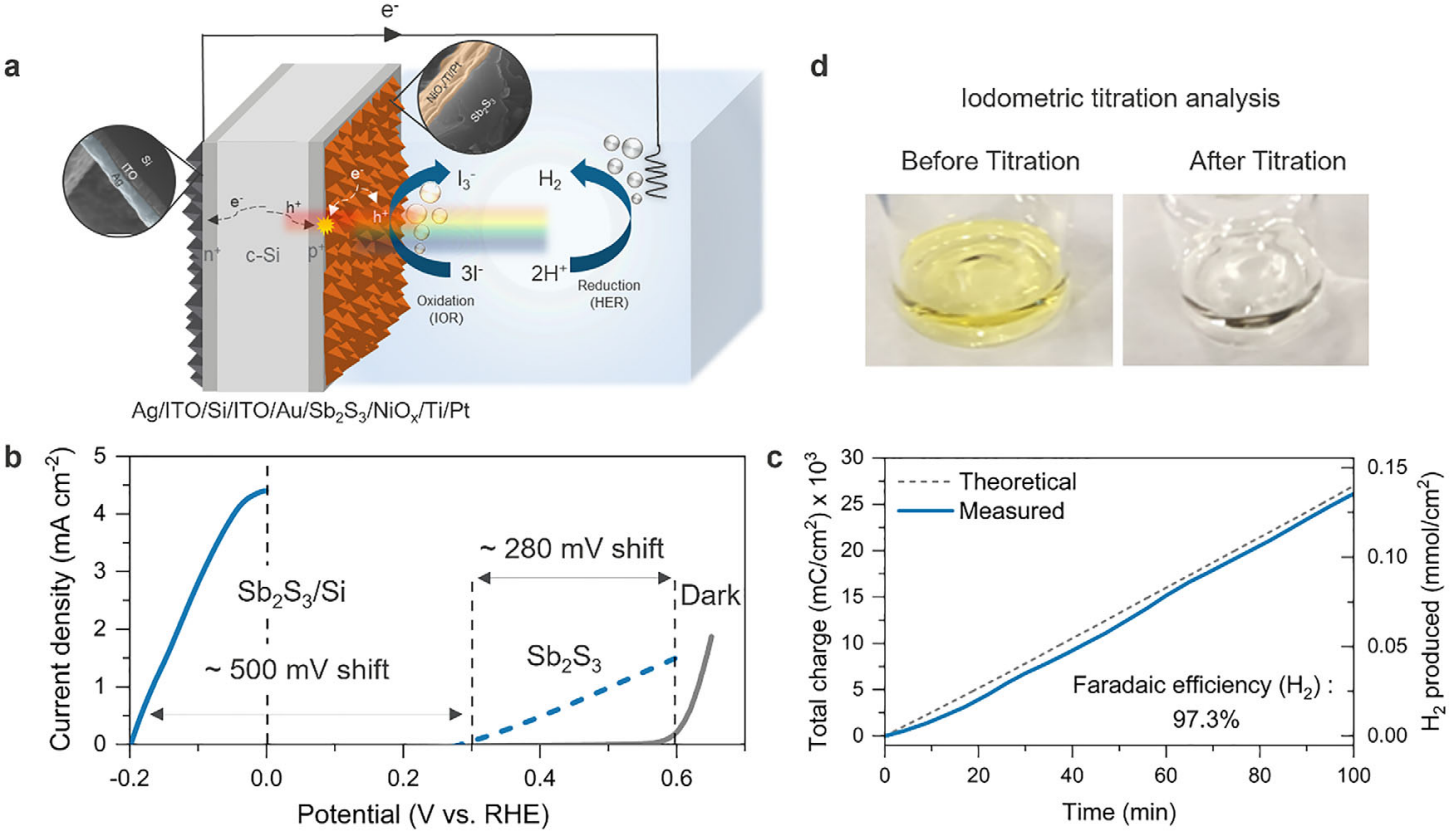
Photoelectrochemical performance and gas evolution analysis of tandem PEC. a) Schematic of the PEC measurement configuration. b) Performance of the tandem PEC device. c) Hydrogen (H_2_) production of the Ag/ITO/Si/ITO/Au/Sb_2_S_3_/NiO_x_/Ti/Pt monolithic tandem device measured under simulated AM 1.5G illumination in a two‐electrode configuration at unbiased condition. The black dotted line denotes the theoretical H_2_ amount calculated from the integrated chronoamperometry charge assuming 100% Faradaic efficiency, while the solid blue line shows the experimentally quantified H_2_ amount measured by gas chromatography. d) Photographs of the anolyte before and after iodometric titration, showing the disappearance of the yellow color after titration with 0.01 M Na_2_S_2_O_3_, consistent with consumption of the oxidized iodine species.

The PEC characteristics of the full monolithic Sb_2_S_3_/Si tandem photoelectrode for the HER‐IOR reaction was analyzed in a three‐electrode configuration under simulated AM1.5G solar illumination (100 mW/cm^2^). We intentionally adopted an externally wired monolithic configuration, as it facilitates rigorous electrochemical characterization while offering practical advantages over fully wireless designs, including reduced solution ohmic resistance through flexible cathode placement and the ability to readily exchange the cathodic catalyst to target diverse reduction reactions beyond HER (see Text S3 for detailed discussion). For a fully wireless design, one proposed solution for the ion transport problem in wireless devices is to perforate the electrode to create additional ionic pathways; however, this approach introduces processing complexity and additional optical losses in the perforated regions where light absorption is sacrificed. Moreover, the inability to apply external bias in wireless configuration precludes electrodeposition of metal catalysts and would inevitably require physical deposition approaches. Figure 6a shows the schematic of the PEC measurements where HER is coupled to IOR at Pt/Ti/NiO_x_/Sb_2_S_3_ surface. In the first linear sweep voltammetry (LSV) scan, the tandem PEC device exhibited an onset voltage of approximately −0.2V vs. RHE and delivered a photocurrent density of 4 mA/cm^2^ at 0 V vs. RHE (Figure S13a). However, a second LSV scan performed on the same device revealed a significant photovoltage decrease of approximately 340 mV, which is due to the degradation (oxidation) of the NiO_x_ layer (Figure S13a). Stability test under unbiased conditions (Figure S13b) similarly revealed rapid device degradation, with the photocurrent density rapidly reducing within tens of seconds. These results clearly indicate that introducing a suitable protective layer on the surface of the top absorber layer is essential for achieving stable and long‐term PEC operation.

Figure 6b presents the LSV of tandem devices with Ti/Pt protective layers measured in a three‐electrode configuration for IOR. The photocurrent density for IOR reaction remains low for FTO/Au/Sb_2_S_3_/NiO_x_ (single cell) photoanode PEC device (<2 mA/cm^2^). In contrast, the tandem PEC device showed significantly enhanced performance, achieving a photocurrent density of 4.4 mA/cm^2^ at 0 V vs. RHE, which directly represents the achievable self‐driven photocurrent density for unbiased solar fuel (H_2_) production. The higher photocurrent density in the case of tandem PEC compared to Sb_2_S_3_ single cell could be rationalized by efficient carrier separation by virtue of Z‐scheme. The onset potential cathodically shifts (toward negative potential along the x‐axis) for the tandem PEC compared to the Sb_2_S_3_ single cell PEC devices due to additional photovoltage from silicon in the tandem device. Notably, an ultrathin layer of Au improves the interface in FTO/Au/Sb_2_S_3_ and Ag/ITO/Si/ITO/Au/Sb_2_S_3_ stack by removing a barrier‐like response in the JV measurements (Figure S16). As evident in Figure 6b, the silicon bottom cells contribute to the photovoltage of around 500 mV for IOR reaction. To estimate photovoltage from the Si bottom cell, we fabricated a Si photocathode and evaluated it under HER conditions in the same electrolyte environment used for the tandem IOR experiments. Given that a single‐cell photocathode based on HIT‐stacked silicon exhibits a photovoltage of around 590 mV (Figure S17), the 500 mV additional photovoltage from the silicon bottom cell clearly demonstrates efficient monolithic integration of the Sb_2_S_3_ and silicon with minimal voltage losses. Slight decrease (∼90 mV) of the photovoltage from silicon in tandem device is partly attributed to reduced light illumination in the tandem configuration, as the incident light is filtered through the Sb_2_S_3_ top cell. Additionally, partial degradation of silicon caused by the Sb_2_S_3_ processing may contribute to this decrease (see also Figure S18). To more definitively quantify the amount of hydrogen in our tandem device, we performed gas chromatography (GC) analysis of our tandem PEC device. As shown in Figure 6c, the Faradaic efficiency of our tandem device for HER was found to be close to 100%, assuming no parasitic contributions to the photocurrent. In addition, the iodine products generated at the photoanode were directly quantified by iodometric titration, which yielded a Faradaic efficiency of 99.84% for the anodic IOR, Figure 6d (see Supplementary Figure S19 for the detailed procedure and analysis). Together, these results confirm that the photogenerated charges are consumed almost exclusively by the targeted HER and IOR half‐reactions.

**FIGURE 7:**
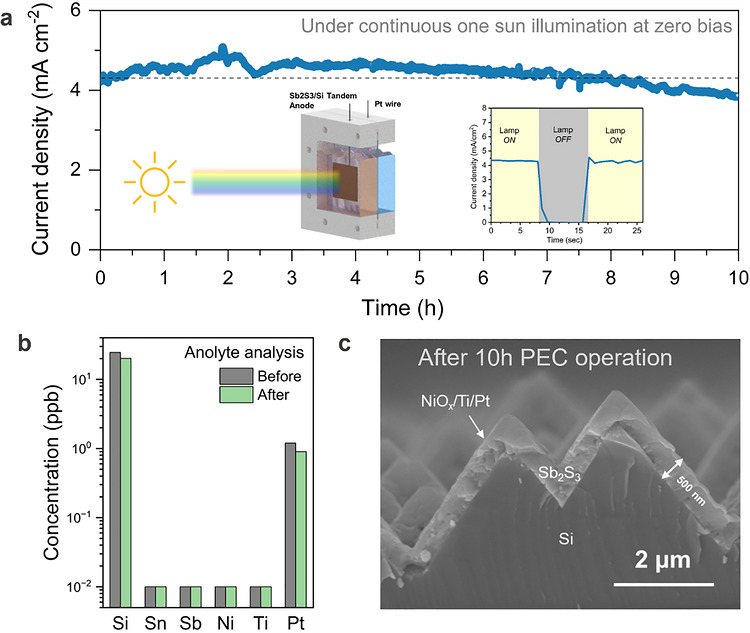
Long‐term stability and post‐operation analysis of tandem PEC. a) Long‐term stability test of monolithic Sb_2_S_3_/Si tandem device without applied bias under 1‐sun illumination. Inset: Two‐electrode chronoamperometry (CA) of the Ag/ITO/Si/ITO/Au/Sb_2_S_3_/NiO_x_/Ti/Pt tandem device under unbiased conditions with chopped illumination, showing a reversible current response upon lamp on/off switching to distinguish photocurrent from dark current. b) Elemental concentration measured before and the long‐term stability test of the unbiased IOR‐HER reaction using Ag/ITO/Si/ITO/Au/Sb_2_S_3_/NiO_x_/Ti/Pt PEC device. c) Cross‐sectional SEM image of the full Sb_2_S_3_/Si tandem stack after 10 h bias‐free operation, showing preserved layer continuity and unaltered Sb_2_S_3_ thickness of ∼500 nm.

To evaluate the unbiased long‐term stability, chronoamperometry (CA) measurements were conducted on the Sb_2_S_3_‐Si tandem PEC device, as shown in Figure 7a. The CA measurement was performed in a two‐electrode configuration consisting of the Sb_2_S_3_‐Si tandem photoanode and a Pt coil cathode under simulated AM 1.5G illumination (1 sun) without any external bias. To prevent backward reactions at the cathode (e.g., reduction of iodine generated at the photoanode), a membrane separating the anolyte and catholyte was included in the PEC reactor. A detailed schematic and description of the PEC reactor are provided in Figures S20 and S21. The Sb_2_S_3_‐Si tandem stack initially exhibited a high unbiased photocurrent density of 4.38 mA/cm^2^ and demonstrated remarkable stability, maintaining 90% of its initial photocurrent density even after 10 h of continuous unbiased operation.

To more rigorously validate the long‐term device stability, we conducted inductively coupled plasma optical emission spectrometry (ICP–OES) analysis of the anolyte before and after the 10‐h stability test (Figure 7b). ICP‐OES directly quantifies any dissolution of constituent elements into the electrolyte, thereby enabling a more stringent assessment of durability for the Sb_2_S_3_/Si tandem stack. Since the anolyte used in this study was based on 0.5 m H_2_SO_4_, accurate quantification of sulfur (S) was challenging due to its inherently high concentration. Therefore, we selectively monitored concentration changes of key constituent elements (Ti, Pt, Si, Sn, Sb, Ni), excluding sulfur. Despite its high sensitivity (typically at the ppb), ICP‐OES detected no measurable Ti or Pt dissolution after 10 h of operation, indicating that the Sb_2_S_3_ layer remained effectively protected by the Ti sealing layer. These results confirm that the Ti and Pt protective layers effectively stabilized the Sb_2_S_3_‐Si tandem structure, even under harsh anolyte conditions containing iodide and strong acid. Meanwhile, the relatively high silicon concentration observed in the ICP–OES measurements is attributed not to degradation of the PEC device, but rather to the leaching of silicon species from the glass vial used for sample storage. To directly evaluate the layer continuity and interfacial integrity of the buried Sb_2_S_3_ layer after operation, we performed cross‐sectional SEM on the sample after 10 h of bias‐free operation. As shown in Figure 7c, the Sb_2_S_3_ layer remains continuous on the textured Si surface without apparent delamination or change in the Sb_2_S_3_ layer thickness in the pristine device. The dissolution analysis (ICP‐MS/ICP‐OES) together with cross‐sectional SEM after operation shows no evidence of corrosion‐driven material loss from the photoanode. Accordingly, under bias‐free PEC operation, the anodic current must be predominantly consumed by the iodide oxidation reaction, consistent with the high Faradaic efficiency observed.

Regarding the solar‐to‐hydrogen (STH) efficiency of the device, the use of an alternative oxidation reaction requires particular care when discussing STH efficiency in the conventional sense. Specifically, the relevant anodic equilibrium potential for the I^−^/I_2_ couple is electrolyte‐dependent: on the RHE scale it increases with pH (because IOR is not proton‐coupled), and it also varies with iodide/triiodide activities (concentration) via the Nernst relation. Therefore, the thermodynamic potential used in an “IOR‐based STH” calculation is not a single fixed value but depends on the electrolyte conditions. Nevertheless, for the sake of clarity and benchmarking, we report our bias‐free operating photocurrent density together with hydrogen Faradaic efficiency as the primary performance metrics for this HER – IOR system. Under unbiased operation, the Sb_2_S_3_/Si tandem delivers 4.38 mA cm^−2^, and GC quantification confirms a near‐unity H_2_ Faradaic efficiency. If one expresses the performance as an *STH‐equivalent* metric, based on the total hydrogen produced per unit time and unit area, referenced to overall water splitting $\left(\eta_{\mathrm{STH},\mathrm{eq}}=J\times 1.23\;V\times FE_{H_{2}}/P_{in}\right)$, 4.38 mA cm^−2^ corresponds to 5.4% (or ∼5.2% using the measured FE). Our study constitutes a major advancement toward ideal monolithic tandem PEC architecture that is cost‐effective and capable of driving versatile chemical transformations.

**FIGURE 8:**
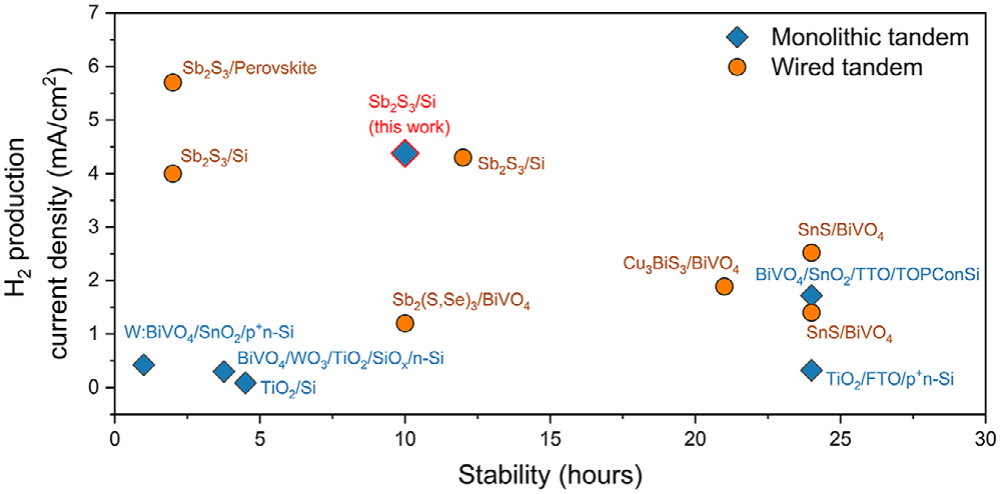
Benchmarking of the present Sb_2_S_3_/Si monolithic tandem PEC device against previously reported systems. Comparison current density and stability (at 0 V_RHE_) of wired/mechanically stacked (circle) and monolithically integrated (diamond) inorganic tandem PEC devices. (Data points taken from the references [4, 17, 21, 22, 55, 56, 57, 58, 59, 60, 61, 62]).

We benchmarked the present Sb_2_S_3_/Si tandem performance against previously reported cost‐relevant, non‐III–V inorganic tandem PEC architectures. This comparison scope was selected to reflect the materials cost and resource availability considerations required for industrial PEC deployment. Even though III–V‐based tandem PEC systems show high efficiencies, are excluded from the direct comparison due to costly epitaxial growth, expensive and scarce raw materials (prices in the range $300–420 kg^−^^1^ for Ga and In) [63, 64], limited wafer scaling, complex device architectures, and lower manufacturing yield compared to silicon. Prior monolithic demonstrations in this cost‐relevant category have thus far been largely dominated by wide‐bandgap oxide/Si architectures, Figure 8. Within this comparison set, the present device delivers the highest reported bias‐free H_2_‐production current density (4.38 mA cm^−^^2^). Although certain oxide‐based monolithic tandems have been operated for longer durations, their photocurrent retention at the end of the stability test was substantially lower: For example, BiVO_4_/SnO_2_/TTO/TOPCon Si device retained only ∼50% of their initial performance, whereas our Sb_2_S_3_/Si tandem still retained ∼92% of its initial photocurrent after 10 h of continuous bias‐free operation. Consistent with this high retained activity, ICP‐OES analysis of the post‐operation anolyte detected no measurable dissolution of photoabsorber constituent elements, further supporting the excellent chemical robustness of the tandem stack. Moreover, to the best of our knowledge, no prior monolithically integrated tandem PEC device based on a chalcogenide photoelectrode has been reported. When compared with wired or mechanically stacked chalcogenide tandems (SnS, Cu_3_BiS_3_), the present monolithic device remains competitive in both H_2_‐production current density and operational stability despite the more stringent integration constraints inherent to monolithic architecture.

## 3 Conclusions

In summary, we have introduced a low‐temperature physical vapor deposition approach to conformally grow highly crystalline Sb_2_S_3_ layer directly on heterojunction (HIT) textured silicon bottom cell and realize a glass‐free monolithic Sb_2_S_3_‐Si tandem PEC device with tailored charge selective contacts. The resulting tandem device showed unassisted and stable photoelectrochemical HER paired with IOR reaction. The band alignment of the functional layers in the Sb_2_S_3_‐Si tandem stack favors a Z‐scheme charge transport, facilitating efficient transport and collection of electrons and holes in the respective layers for utilization in the electrochemical reaction at the electrolyte interface. We demonstrated the feasibility of the tandem PEC devices for IOR reaction, coupled with HER for enhanced performance. The optimized monolithic Sb_2_S_3_–Si tandem PEC device with Ti/Pt protection and catalyst layer, demonstrated conversion of H^+^ and I^−^ to H_2_ and I_3_^−^ with photocurrent density of 4.38 mA cm^−^^2^ at 0 V vs. RHE and excellent stability (retaining approximately 90% of its original photocurrent after 10 h of continuous operation) under AM 1.5G simulated illumination. Our work provides an important step toward artificial photosynthesis systems through monolithic integration of ideal bandgap photo‐absorbers in tandem configuration by cost‐effective and high‐volume synthesis.

## Author Contributions

Sudhanshu Shukla, Bart Vermang, and Byungha Shin designed and managed the project. Sudhanshu Shukla conceptualized the idea, developed the tandem PEC stack and designed the experiment. Sudhanshu Shukla, Irene Dei Tos, Jihong Min, and Devika Rajagopal synthesized the photoelectrodes. Jihong Min conducted the PEC performance evaluation. Sudhanshu Shukla, Jihong Min, Irene Dei Tos, Jan D'Haen, Beatriz de la Fuente, Sepideh Rahimisheikh conducted characterizations. Sepideh Rahimisheikh and Joke Hadermann conducted and analyzed transmission electron microscopy results. Beatriz de la Fuente and Tom Hauffman conducted and analyzed photoelectron spectroscopy. David Cornil and David Beljonne build computational model and its analysis. Sudhanshu Shukla and Jihong Min drafted the manuscript. Sudhanshu Shukla, Jihong Min Beatriz de la Fuente, Irene Dei Tos, Jan D'Haen, Sepideh Rahimisheikh, Joke Hadermann, Tom Aernouts, Bart Vermang, and Byungha Shin contributed to the discussion, revision and completion of the manuscript. Sudhanshu Shukla, Bart Vermang, and Byungha Shin supervised the work.

## Conflicts of Interest

The authors declares no conflicts of interest.

## Supporting information




**Supporting File**: advs75798‐sup‐0001‐SuppMat.docx.

## Data Availability

The data that support the findings of this study are available from the corresponding author upon reasonable request.
