# Supplementary material for: Fully Textured Monolithic Sb2S3/Silicon Tandem for Unbiased and Stable Solar‐Driven Water Splitting Paired with Iodide Oxidation Reaction
Source: Adv Sci (Weinh). 2026 May 22:e75798. Online ahead of print. doi: 10.1002/advs.75798 (PMC13335926; doi:10.1002/advs.75798)
Supplement: Supplementary file 1 — Supporting File: advs75798‐sup‐0001‐SuppMat.docx. [file ADVS-9999-e75798-s001.docx]

Supplementary Information

**Fully textured monolithic Sb_2_S_3_/Silicon tandem for unbiased and stable solar-driven water splitting paired with iodide oxidation reaction**

*Jihong Min^1^, Irene Dei Tos^2,3,4^, Sepideh Rahimisheikh^5^, Beatriz de la Fuente^6^, Devika Rajagopal^2,3,4^, Jan D’Haen^7,8^, David Cornil^9^, Tom Hauffman^6^, Tom Aernouts^2,3,4^, David Beljonne^9^, Joke Hadermann^5^, Byungha Shin^1^*, Bart Vermang^2,3,4^, Sudhanshu Shukla^2,3,4^**

^1^ Department of Materials Science and Engineering, Korea Advanced Institute of Science and Technology, Daejeon 34141, Republic of Korea

^2^ imec, IUMAT, Thor Park 8320, B-3600 Genk, Belgium

^3^ UHasselt, Institute for Materials Research (IUMAT), Martelarenlaan 42, B-3500 Hasselt, Belgium

^4^ EnergyVille, Thor Park 8320, 3600 Genk, Belgium

^5^ University of Antwerp, Electron Microscopy for Materials Science (EMAT), Antwerpen 2020, Belgium

^6^ Research Group Sustainable Materials Engineering (SUME), Lab of Electrochemical and Surface Engineering (SURF), Vrije Universiteit Brussel, Pleinlaan 2, Brussels 1050, Belgium

^7^ UHasselt, Institute for Materials Research (IUMAT), Analytical & Microscopical Services (AMS), Martelarenlaan 42, B-3500 Hasselt, Belgium
^8^ imec, IUMAT, Wetenschapspark 1, B-3590 Diepenbeek, Belgium

^9^ Laboratory for Chemistry of Novel Materials, University of Mons (UMONS), 20 Place du Parc, 7000 Mons, Belgium

*Corresponding authors: sudhanshu.shukla@imec.be and byungha@kaist.ac.kr

**Text S1. Experimental details**

**Deposition of Sb_2_S_3_ thin films**

The silicon substrates were placed in the vacuum evaporation chamber with 150 mg of Sb_2_S_3_ powder (Sigma-Aldrich) with a base pressure of 10^-5^ mbar. The temperature of the crucible was increased with a ramp rate of 17^o^C/minute until it reached 530 °C. Thermal evaporation took place at 530 °C for 30 seconds while the substrate was held at room temperature. Subsequently, the chamber was cooled down to room temperature (RT). Once RT has been reached, the samples were taken out and annealed in a two-zone tube furnace. The samples were placed on the right zone of the quartz tube, while the other zone was occupied by a quartz crucible containing 100 mg of sulfur powder (Sigma-Aldrich). The samples were annealed at 335 °C for 15 minutes using a ramping rate of 10 °C/min, while the sulfur powder was heated up to 200 °C. Once the process was finished, the samples were slowly cooled down to RT inside the tube furnace.

**Si heterojunction solar cell fabrication**

N‐type, 170μm thick, 156 x 156 mm^2^, square, ~2.5 Ω.cm, (100) Cz Si wafers were used for device fabrication. Our baseline process flow is depicted in Figure S1. The wafers were textured with the KOH and an additive monoTEX^®^ H2.0 from RENA Technologies Gmbh to achieve pyramids of height 2.50±0.65 mm with weighted reflectance *R_W_* (300 – 1200 nm) of 17.8±0.1 % (see Figure S1). The textured wafers were cleaned using imec’s industrial cleaning process involving deionised water (DIW):HF:O_3_. An additional clean using a sulphuric acid-peroxide mixture (SPM), *i.e.*, H_2_SO_4_:H_2_O_2_ (4:1) helped to remove any organic contamination on the wafer surface, *e.g.*, due to storage in a wafer box between interleaves. The mixture is a strong oxidising agent and removes the organic contaminants by destroying the hydrocarbon bonds. Prior to depositions, the wafers are dipped in 1% dilute HF solution to remove the chemically grown oxide. A stack of i-p^+^ a-Si:H was deposited at the front surface while a stack of i-n^+^ a-Si:H was deposited at the rear using PECVD (temperature of 200°C, an RF power density of 25 mW/cm^2^ and a pressure of 2.3 mbar). The wafers resulted in an effective lifetime of 3ms at the injection level Δn = 10^15^ cm^-3^ after passivation with an implied Voc of [fill up]. Indium tin oxide (ITO) as a transparent conductive oxide (TCO) was then sputtered at the front.

**Deposition of layers in the tandem stack**

For Ti, Pt: The Ti protection layer and Pt electrocatalyst were deposited by electron-beam evaporation. The nominal thicknesses of Ti and Pt were set to 10 nm and 2 nm, respectively. The deposition was carried out at a rate of 0.4 nm s⁻¹.

Au and Ag: Au and Ag layers were deposited by thermal evaporation. The nominal thicknesses of Au and Ag were set to 10 nm (rate 2 Å s^-1^) and 100 nm (rate 1 Å s^-1^) respectively.

ITO layer was deposited using RF sputtering process with 25% power (750 W), working pressure of 300 mTorr and O_2_ and Ar flow rates of 0.125 sccm and 19.875 sccm respectively. Thickness was controlled by varying the number of passes. The thickness of ITO recombination layer at the Si-Sb_2_S_3_ interface was set to ~ 30 nm (3 passes). Back ITO contact layer thickness was set to ~ 100 nm (10 passes).

NiO_x_: NiO_x_ was deposited using RF magnetron sputtering from NiO target in a Nebula system from Angstrom Engineering with a base pressure of ~10^-8^ Torr, designed by Angstrom Engineering Inc. A mixture of 15 sccm Ar and 0.3 sccm O_2_ gas was used for the plasma at 3 mTorr. RF sputtering was adopted for the deposition of NiO_x_ as HTL. This deposition process, while presenting the advantage of avoiding the standard annealing step at ~300 °C, can render the NiO_x_ films less conductive. Thus, the thickness of NiOx layer was optimized and set to 30 nm to render it an HTL without obstructing the passages of holes to the surface of the photoanode.

**Scanning electron microscopy (SEM)**

Scanning electron microscopy (SEM) measurements of the tandem stack were carried out on a Zeiss 450 Gemini 2 FEG-SEM. SEM images of the textured silicon were acquired by Nova 200, FEI field-emission SEM under an accelerating voltage of 3 kV.

**Transmission electron microscopy (TEM)**

To study the cross-section of the tandem, a FIB lamella was prepared on a Cu Omniprobe TEM grid, using a Thermo Fisher Helios FIB-SEM. The sample was loaded on a FEI standard double tilt TEM holder in ambient conditions and transferred to the microscope. For the characterization, High Angle Annular Dark Field Scanning Transmission Electron Microscopy (HAADF-STEM) and Energy Dispersive X-ray spectroscopy (EDX) were performed on an aberration-corrected Thermo Fisher Titan transmission electron microscope at 300 kV, using a Super X detector. The TEM-EDX map was acquired over a 10-minute exposure with drift correction.

Orientation analysis: The analysis is done using a Thermo Fisher Tecnai G2 TEM at 200 kV equipped with the “ASTAR” tool (automatic TEM phase-orientation mapping) and ASI Timepix3 Direct electron detector. To obtain the phase map, we oriented the FIB sample to the [110] zone axis of the silicon substrate, which is the closest main zone accessible in this sample. Then, a region of the sample is scanned by the electron beam (a semi-parallel microprobe with approximately 3nm probe size) at a step size of approximately 0.5 nm for 1 ms, and the diffraction pattern is recorded on the detector simultaneously. The total time of acquisition per scan is about 4-5 minutes. The post-processing is done using the software DiffGen (ACOM-SIMAP NanoMEGAS SPRL version 2.0.10.960), Index (ACOM-SIMAP NanoMEGAS SPRL version 2.0.10.1552), Mapviewer (ACOM-SIMAP NanoMEGAS SPRL version 2.0.10.474) and OIM Analysis (EDAX version 8.0). The OIM Analysis software is used for cropping and cleaning the final map. The maps are cleaned with the grain dilation method with a grain tolerance angle of 10 degrees and a minimum grain size of 40 pixels. Since the non-standard symmetries were not acceptable in the “DiffGen” software to make the data bank, the Pbnm space group of Sb_2_S_3_ transformed to Pnma. The axes transform as follows:

$$a_{Pbnm}= c_{Pnma}$$

$$b_{Pbnm}= a_{Pnma}$$

$$c_{Pbnm}= b_{Pnma}$$

First, a databank is generated from the CIF files of Silicon (ICSD collection code 51688) and Sb_2_S_3_ (ICSD collection code 30779 transformed into Pnma space group). Then the calibration for the camera length and distortions was done on the silicon region, and later the orientation map of both (substrate and coating) was exported separately.

**X-ray diffraction (XRD)**

Thin film XRD were carried out with a Bruker D8 Advance equipped with a Lynxeye detector and operated with Cu-Kα X-ray (λ = 1.5418 Å) radiation.

**Raman spectroscopy**

A laser excitation of 532 nm with a power of 10 mW and spot size of 1 µm was used for the measurements. The signal was recorded using 20 s acquisition time and 30 accumulations and Olympus 50X long working distance objective.

**X-ray photoelectron spectroscopy (XPS) characterization**

The chemistry and oxidation states of the samples were studied using a VersaProbe III X-ray photoelectron spectroscope (XPS) operating with an Al Kα anode and a main chamber pressure of 5 × 10^−7^ Pa. XPS surveys and high-resolution scans of the Sb 3d and S 2p photoelectron peaks were recorded with an angle of 45° with respect to the sample normal. The selected pass energy was 280 eV and 26 eV and the step size 1 eV and 0.1 eV for the surveys and high-resolution scans, respectively. Ultraviolet photoelectron spectroscopy (UPS) was also performed using the VersaProbe III spectroscope with a He(I) (21.22 eV) light source (wavelength of 58.4 nm). To ensure proper electrical contact, the samples were mounted using copper tape and a 20 mm molybdenum metallic shield with a 5 mm diameter punched hole, provided by the manufacturer. The holder used was a 25 mm one, allowing the sample tilting that is required for UPS. Because of the positioning of the UPS source within the equipment, a correction must be made via the mechanical tilting of the holder to obtain the most counts and thus maximize photoemission. With a 45° tilt with respect to the stage angle used for XPS, the stage becomes perpendicular to the analyzer column. A negative potential of -5 V was provided to the samples to reduce the surface charging and to separate the secondary edges of the sample and analyzer. The sample bias moves the secondary electron emission edge to a kinetic energy, where it is accessible with the energy analyzer (above zero in kinetic energy). A sputter-cleaned Ag sample was used to calibrate the binding energies attained for UPS. This gives a reference where the analyzer Fermi edge is on the energy scale. The sample is then connected to the analyzer by mounting it on the sample plate. This immediately equilibrates the semiconductor Fermi level with that of the analyzer, *i.e.,* the *internal* kinetic energy

spectrum of the semiconductor becomes calibrated, and absolute values for valence band maximum binding energy can be extracted. The determination of the electron cutoff (E_cutoff_), valence band maximum (E_VBM_), and Fermi level (E_F_) was conducted with the Edge tool from the PHI MultiPak software. The E_cutoff_ and E_VBM_ values were extracted by using Multipak’s edge tool that linearly fits the spectrum and intercepts the x-axis with the created fit. From these parameters, the WF and IP values were calculated. The values were computed using the Edge tool function from the Multipak software, and the formulas are shown below, where hv is the energy of the source (21.22 eV).

$W_{F}=h\nu-\left( E_{cutoff}-E_{F} \right)$ (1)

$IP=h\nu-(E_{cutoff}-E_{VBM})$ (2)

Gas Cluster Ion Beam (GCIB) was performed prior to XPS and UPS primarily for surface cleaning, ensuring accurate and representative spectroscopic analysis of the material’s surface. The GCIB settings used were a beam voltage of 20 kV, a target current of 40 nA, and a raster size of 2 mm x 2 mm. These settings produce equivalent cluster sizes of 2500 Ar ions. The sputtering time was 30 s. The respective spot sizes of XPS and UPS were 100 μm and 1.5 mm.

**Photoelectrochemical measurements**

The fabricated photoelectrodes were characterized using photoelectrochemical (PEC) methods in a conventional three-electrode setup employing a quartz cell connected to a potentiostat (Bistat, Biologic Science Instruments). For the counter electrode, a platinum coil was used, while an Ag/AgCl electrode (3 M KCl) served as the reference electrode. Potentials measured versus Ag/AgCl were converted to the reversible hydrogen electrode (RHE) scale using the following equation:

$E_{RHE}=E_{Ag/AgCl}+0.197+\left( 0.0591*V*pH \right)$ (3)

Simulated solar illumination was provided by a 300 W xenon arc lamp (Newport, model 66902) combined with an AM 1.5G filter. The intensity of the incident light was calibrated to 100 mW cm⁻² prior to measurements using a reference silicon photodiode (Newport, model 91150 V).

Electrochemical measurements were performed in an aqueous sulfuric acid electrolyte (0.5 M H_2_SO_4_, pH ≈ 0). For iodide oxidation reaction (IOR) experiments, the electrolyte solution was modified by adding 0.1 M potassium iodide (KI). Linear sweep voltammetry (LSV) and cyclic voltammetry (CV) were carried out, beginning from the open-circuit potential (OCP) and scanning toward anodic potentials at a scan rate of 20 mV s⁻¹.

**Iodine quantification by iodometric titration.**

The amount of iodine generated during the unbiased iodide oxidation reaction was quantified by iodometric titration using sodium thiosulfate as the titrant. After 3 h of bias-free PEC operation, the anolyte was collected and titrated with 0.01 M Na₂S₂O₃ solution until the yellow color of the electrolyte disappeared completely, indicating consumption of the oxidized iodine species. The iodine amount was determined based on the titration reaction,

$$I_{2}+2S_{2}O_{3}^{2-}\to2I^{-}+S_{4}O_{6}^{2-}$$

and the Faradaic efficiency for iodine generation was obtained by comparing the charge calculated from the titration with the total charge passed during unbiased PEC operation.

**Theoretical description of Sb_2_S_3_/oxide interfaces**

The calculations were carried on at the periodical density functional theory (DFT) level using the Vienna Ab initio Simulation Package (VASP) code.^1,2^ The PAW (projector-augmented-wave) formalism was used with a 500 eV cutoff for the energy. Electronic exchange and correlation were considered under the PBE functional.^3^ A Γ-centered Monkhorst-Pack grid of (3 × 3 × 1) k-point was used for interface calculation for both relaxation and computation of electronic properties. A Hubbard correction (DFT+U) was applied to metal elements in oxides (U_eff_ = 7.0 eV for *d* orbitals of Ni and In). The Sb_2_S_3_ slab was built from a pre-optimized bulk. We selected the (001) facet with final lattice vectors along the orthogonal surface plane a = 11.17 Å and b = 11.71 Å. One major issue when describing interface between two slabs is the lattice commensurability *i.e.* the matching error between the lattice vectors of the two interacting slabs. The commensurability is quite poor for the considered oxides with matching error higher than 10% in some directions. Imposing the matching would yield large tensile or compressive strains, possibly biasing the results. To overcome this issue, we instead adopted a cluster-to-slab approach where a finite 0D cluster of the oxide is deposited on top of the Sb_2_S_3_ slab. The geometric structure of the ‘cluster-on-Sb_2_S_3_’ was then allowed to relax prior to electronic structure calculations. Nickel oxide was described by a cubic Ni_32_O_32_ cluster with 001 facet in contact with the Sb_2_S_3_ surface and an anti-ferromagnetic ordering for Ni atom along the (111) cluster direction. For indium oxide, we used a In_14_O_21_ cluster that mimics amorphous ITO.

From the relaxed geometry of the Sb_2_S_3_/oxide interfacial models, we have extracted the projected density of states (PDOS), which provides a direct hint into the relative position of the frontier energy levels of the two layers in contact.

**Text S2. Photoelectrochemical systems : Design and configurations**

Photoelectrochemical (PEC) systems exhibit a fundamental advantage over photovoltaic-electrochemical catalyst separated (PV+EC) systems, in that PEC devices inherently avoid ohmic losses associated with lateral charge transport through TCO layers. In PEC systems, photoexcited charge carriers generated in the semiconductor are utilized directly at the semiconductor-electrolyte. In contrast, PV+EC systems rely on lateral conduction of photoexcited charge carriers through large-area TCO layers to reach the electrocatalyst. Since TCO layers intrinsically possess high sheet resistance, significant ohmic losses inevitably occur due to lateral charge transport. Particularly when scaling devices to practical sizes (>10 cm²), the increased lateral transport distance exacerbates ohmic losses, causing severe reductions in device efficiency. To mitigate these ohmic losses, incorporating a metal grid (e.g., Ag grid) onto the TCO surface has been proposed. However, the introduction of an Ag grid inevitably results in optically inactive regions due to its low optical transmittance, thereby reducing the active device area. Thus, employing an Ag grid offers only a partial solution and does not fundamentally resolve the issue of ohmic losses.

To realize an ohmic-loss-free tandem PEC device—completely eliminating the need for lateral TCO contacts-two stringent conditions must be simultaneously satisfied. The first condition is the monolithic integration of the top and bottom absorbers. Non-monolithic tandem configurations (wired or mechanically stacked) inherently require separate TCO layers for each sub-cell to enable electrical connection and optical integration, inevitably introducing lateral current transport. In contrast, a monolithically integrated tandem PEC device requires only a thin TCO layer (recombination layer) between the sub-cells. Within the recombination layer, charge carriers cover very short distance (on the order of a few tens nanometers), inherently eliminating ohmic losses. The second condition is that the wide-bandgap top absorber layer must directly contact the electrolyte.

**Text S3. Practical advantages of the wired monolithic tandem configuration**

The wired monolithic configuration was a deliberate design choice, as it offers significant practical advantages over a wireless monolithic configuration. In any PEC device, the anode and cathode form a complete electrochemical circuit through the electrolyte, and ion transport between the two electrodes is essential for device operation. In a wireless configuration, particularly at the large-area scales relevant for practical deployment, the ion migration distance increases substantially, leading to significant ohmic losses in the electrolyte. One proposed solution for the ion transport problem in wireless devices is to perforate the electrode to create additional ionic pathways; however, this approach introduces processing complexity and additional optical losses in the perforated regions where light absorption is sacrificed. In contrast, the wired configuration can effectively mitigate these ohmic losses owing to the greater flexibility in cathode placement—for example, positioning the catalyst adjacent to the photoanode—thereby substantially reducing the ion transport resistance.

A further advantage of the wired configuration is that the cathodic catalyst is decoupled from the monolithic absorber stack, enabling facile exchange of the cathode to target different reduction reactions. For instance, by replacing the HER catalyst with Cu-based catalysts (e.g., Cu, Cu_2_O, or CuAg alloys),^4,5^ the same monolithic photoanode platform could be used for solar-driven CO_2_ reduction to produce value-added carbon products such as CO, formate, methanol, ethylene, and ethanol. In a fully integrated wireless PEC device, changing the cathodic reaction would require complete re-fabrication of the entire device, limiting the versatility of the platform. The wired monolithic architecture therefore combines ohmic-loss-free internal charge collection within the monolithically integrated absorber stack with a modular cathodic interface, offering a versatile platform for exploring diverse solar-to-chemical conversion pathways

\

**Fabrication of the textured heterojunction silicon bottom cell**


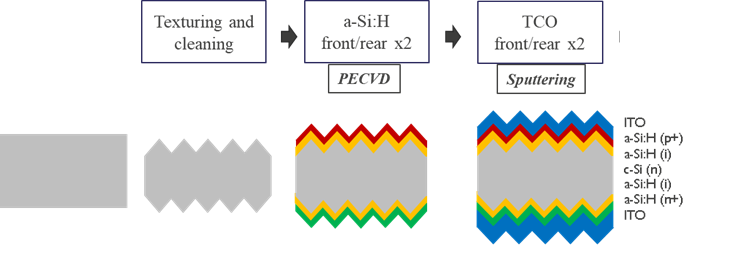


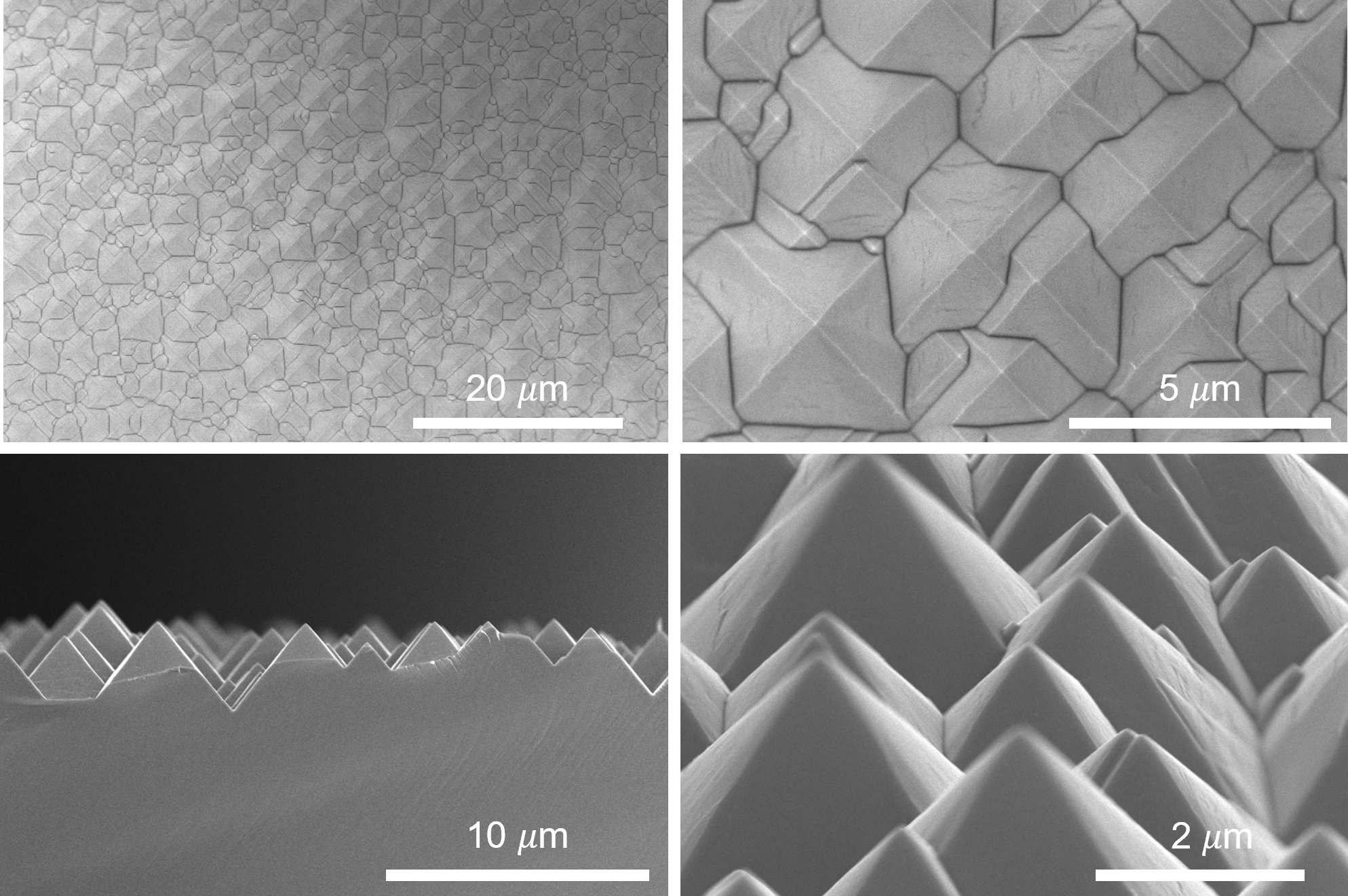


**Figure S1**. The baseline silicon heterojunction front and rear contacted (SHJ) process flow. Top-view, cross-section and tilt-view SEM of the textured silicon substrate.


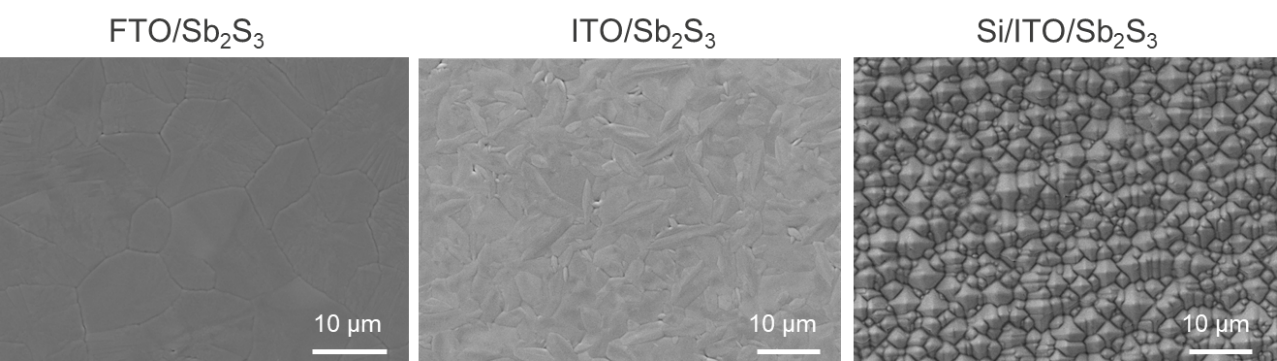


**Figure S2**. Top-view SEM of the Sb_2_S_3_ film deposited on different substrates, (i) planar FTO-coated glass substrates, (ii) planar ITO-coated glass substrates, and (iii) textured silicon substrate.


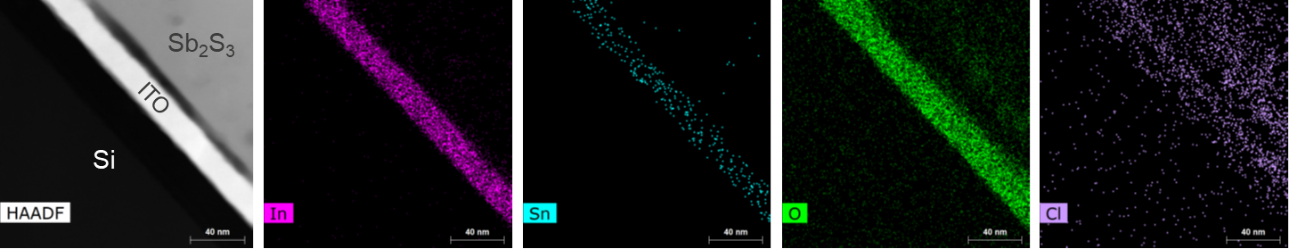


**Figure S3**. HAADF-STEM image and EDX map on the FIB cut lamella for Si/ITO/Sb_2_S_3_ layers.


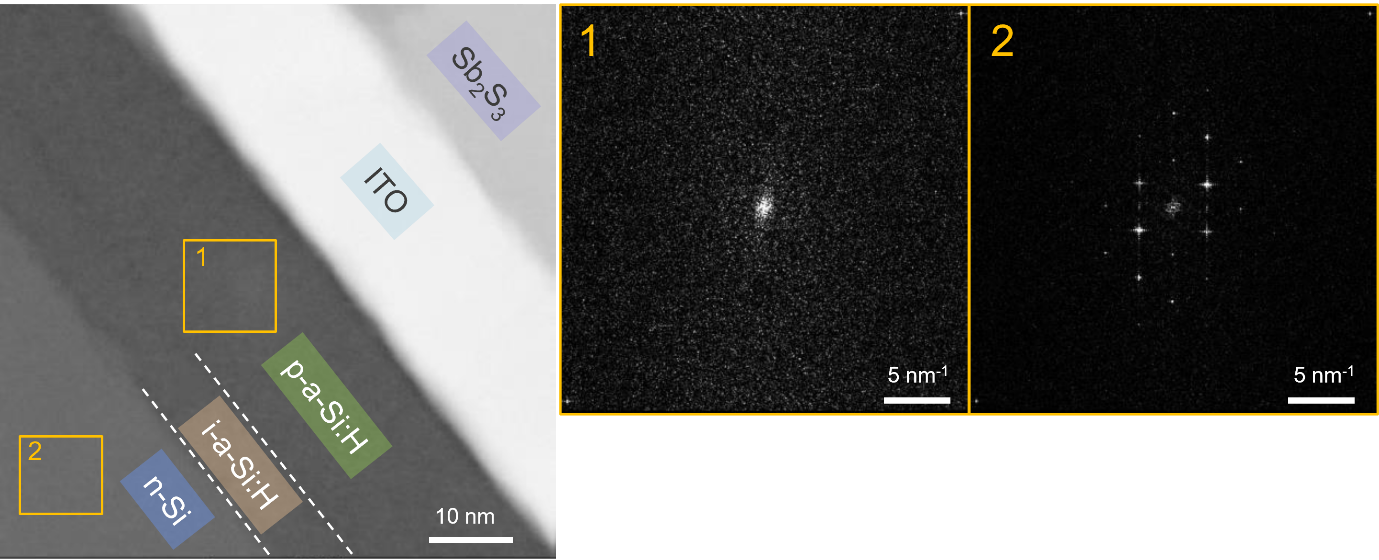


**Figure S4**. Cross-section STEM images of Si/ITO/Sb_2_S_3_ showing n-i-p+ (n-Si/i-a-Si:H/p-a-Si:H) and corresponding FFT patterns of the corresponding marked areas. In the dark band, the FFT of area 1 resembles an amorphous structure (dark band extended to ~ 30 nm). The FFT of area 2 in the n-Si region shows a crystalline pattern.


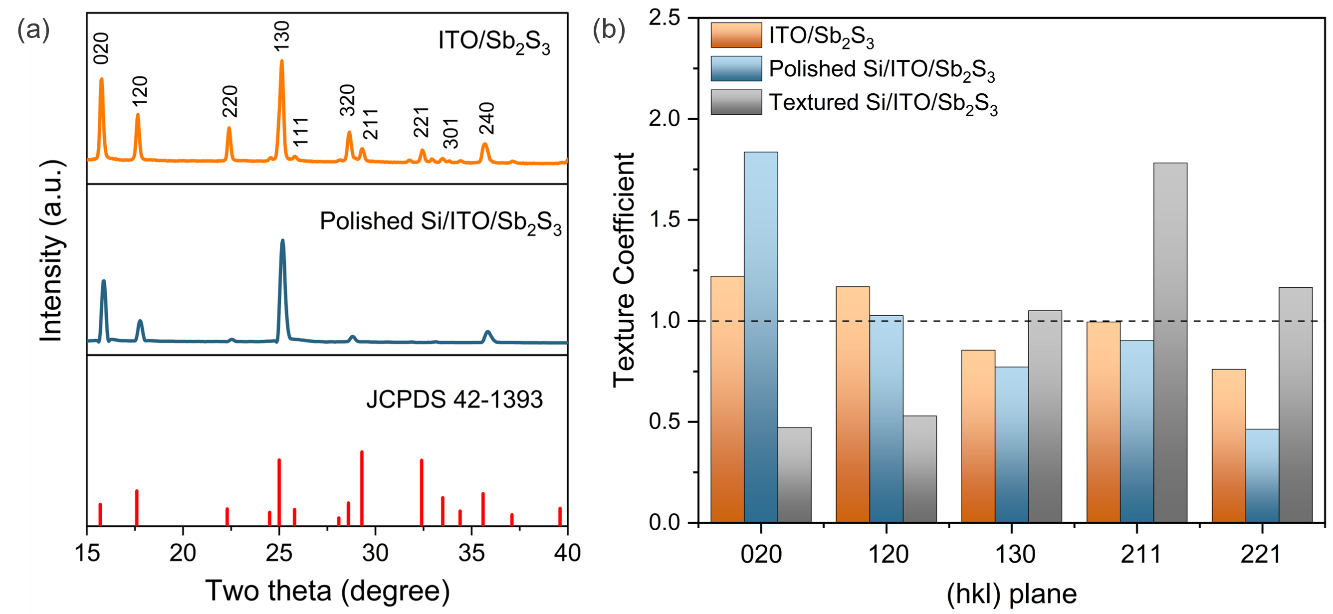


**Figure S5**. (a) X-ray diffraction (XRD) of the Sb_2_S_3_ thin film deposited on planar ITO-coated glass substrate and polished ITO-coated silicon substrates. (b) Texture coefficients of the dominant reflection planes derived from the XRD.


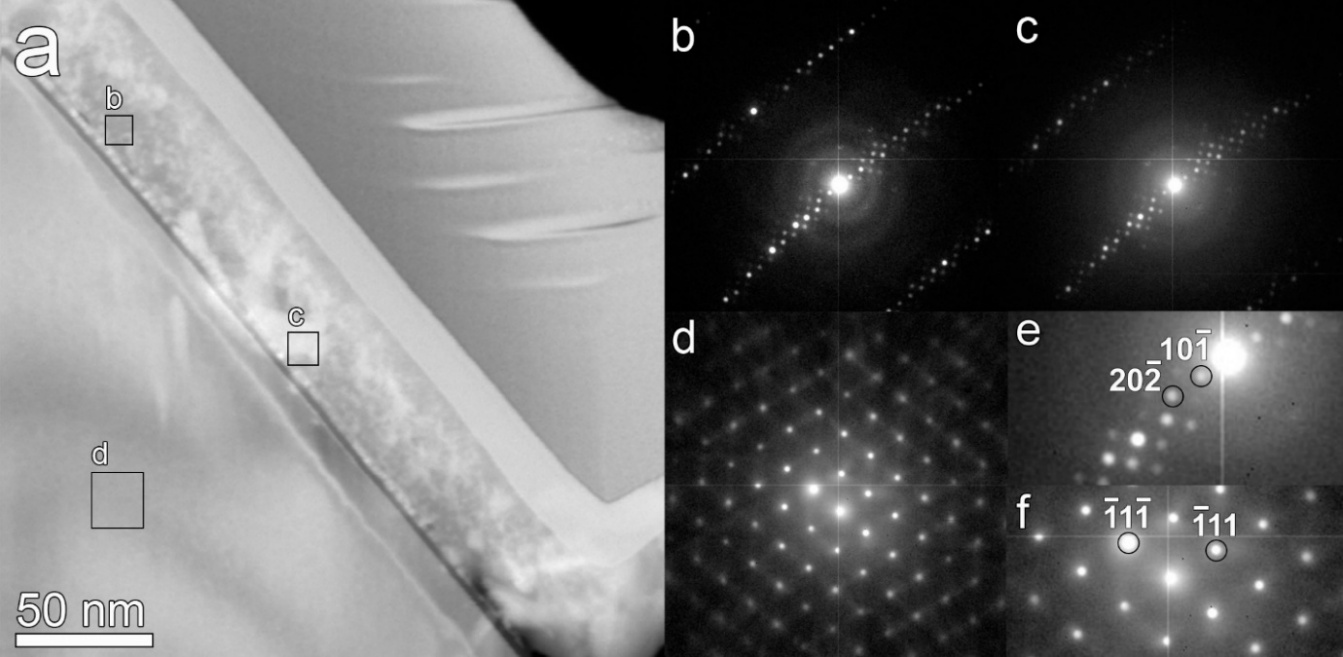


**Figure S6**. (a) 4D STEM scanned image, (b-d) integrated diffraction patterns of the selected pixels indicated on a by rectangles, (e and f) enlarged diffraction pattern around the central beam from c and d, respectively. Sb_2_S_3_ is indexed with Pnma space group.(note that $a_{Pnma}= b_{Pbnm}$, $b_{Pnma}= c_{Pbnm}$, $c_{Pnma}= a_{Pbnm}$)

Figure S6 shows the scanned area of the sample and the diffraction patterns, each of which is an integration of several diffraction patterns obtained from the regions indicated on figure (a) by black rectangles. Figures S6-b and S6-c are from the coating layer, which shows a similar orientation of the grains. In the magnified images (Figures S6-e and S6-f), the patterns for the coating and the substrate are indexed according to the template matching, respectively. This shows that there is no direct relation between the orientation of the substrate (110-zone) and the coating.


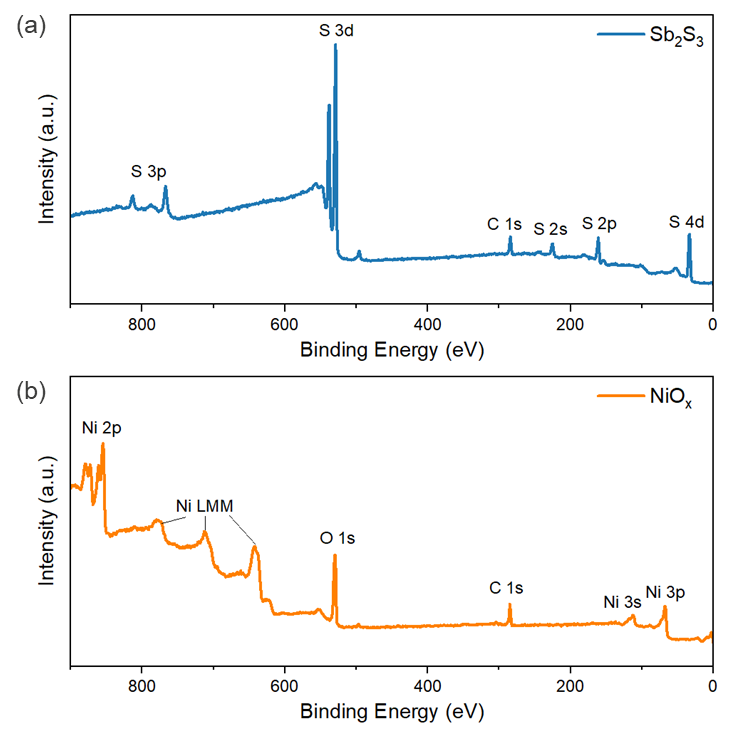


**Figure S7**. XPS survey scan for Sb_2_S_3_ and NiO_x_ film.


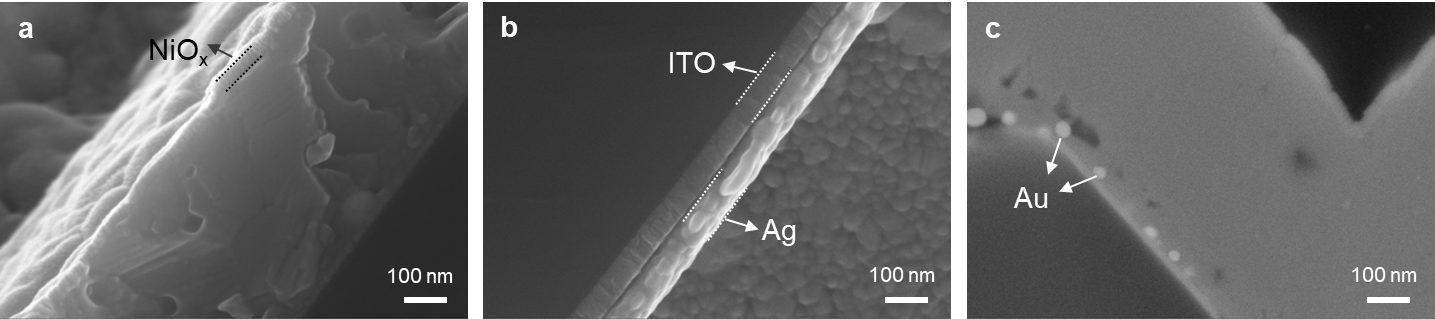


**Figure S8.** Cross-section SEM image of (a) 30 nm NiO_x_ on Sb_2_S_3_ at the front (top) surface of the photoanode, (b) 80 nm ITO and 100 nm Ag layers on Si at the back (bottom) surface of the photocathode, and (c) 10 nm Au at the ITO recombination layer interface.


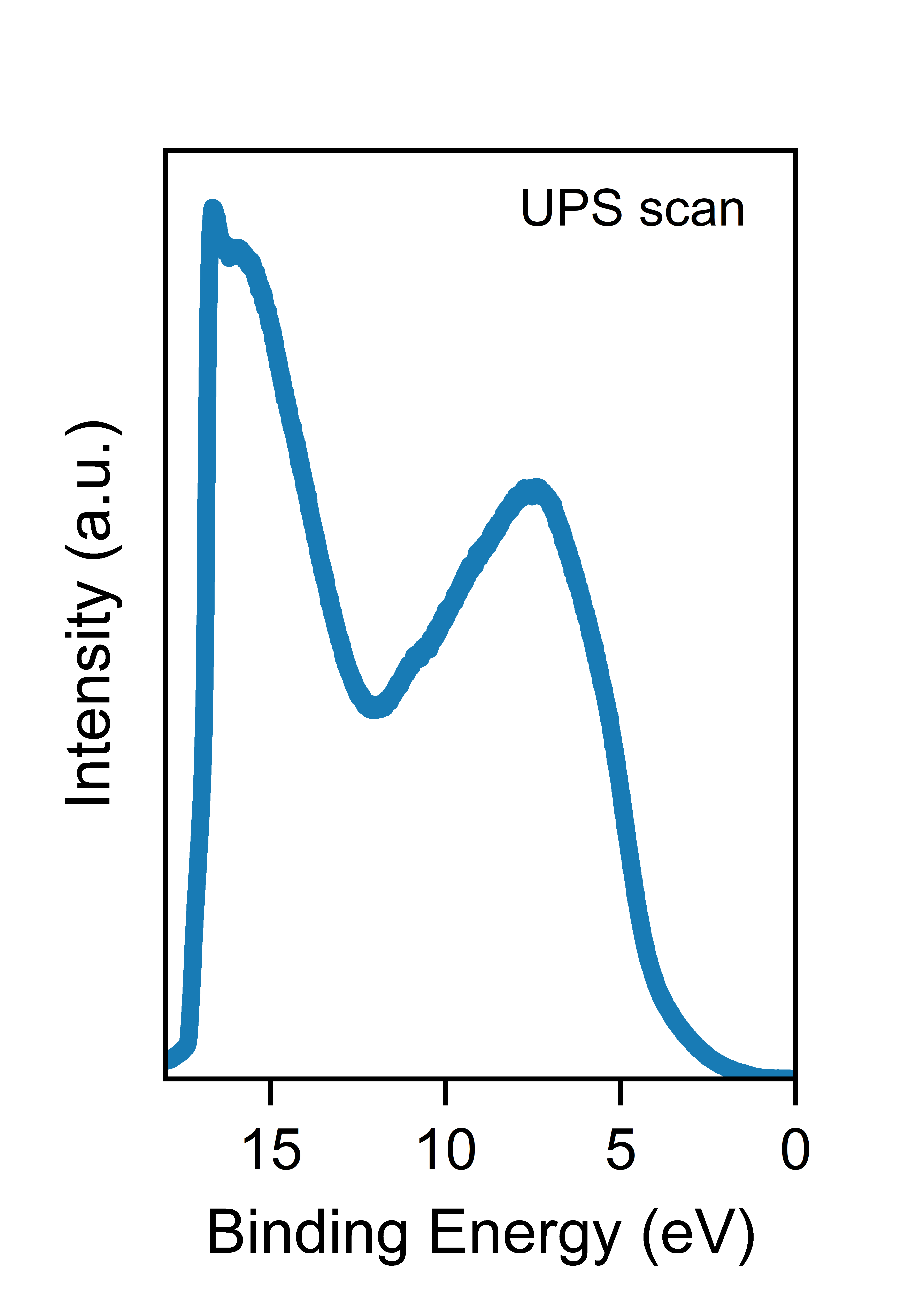


**Figure S9.** Full UPS scan of Sb_2_S_3_ film.


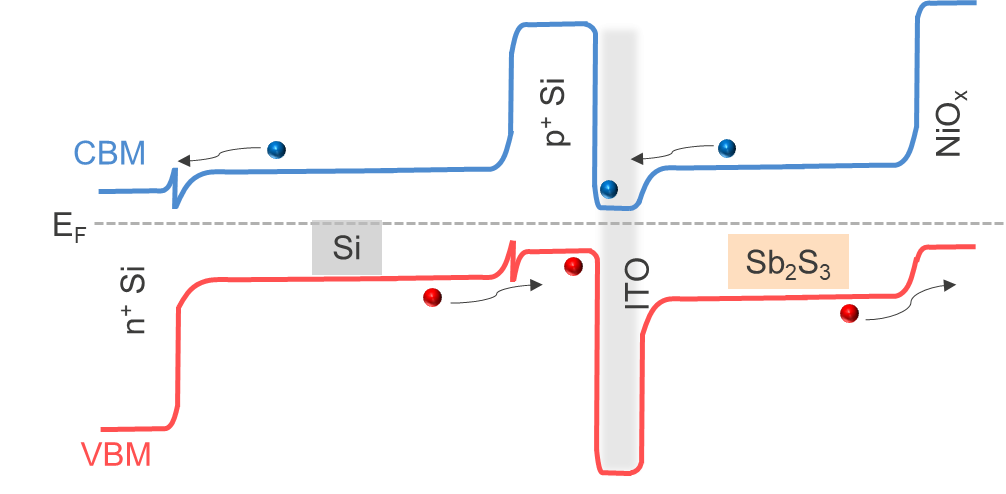


**Figure S10**. Energy band diagram of the full tandem device.


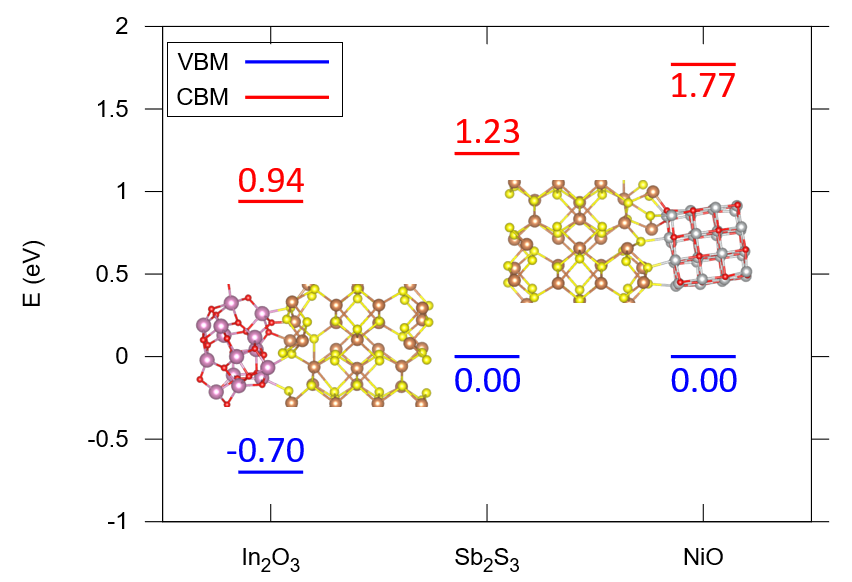


**Figure S11.** Relative position of the frontier levels (valence band maximum, VBM and conduction band minimum, CBM) based on the projected density of states computed for In_2_O_3_/Sb_2_S_3_ and NiO/Sb_2_S_3_ interfaces (with a side view of the two optimized interfaces).


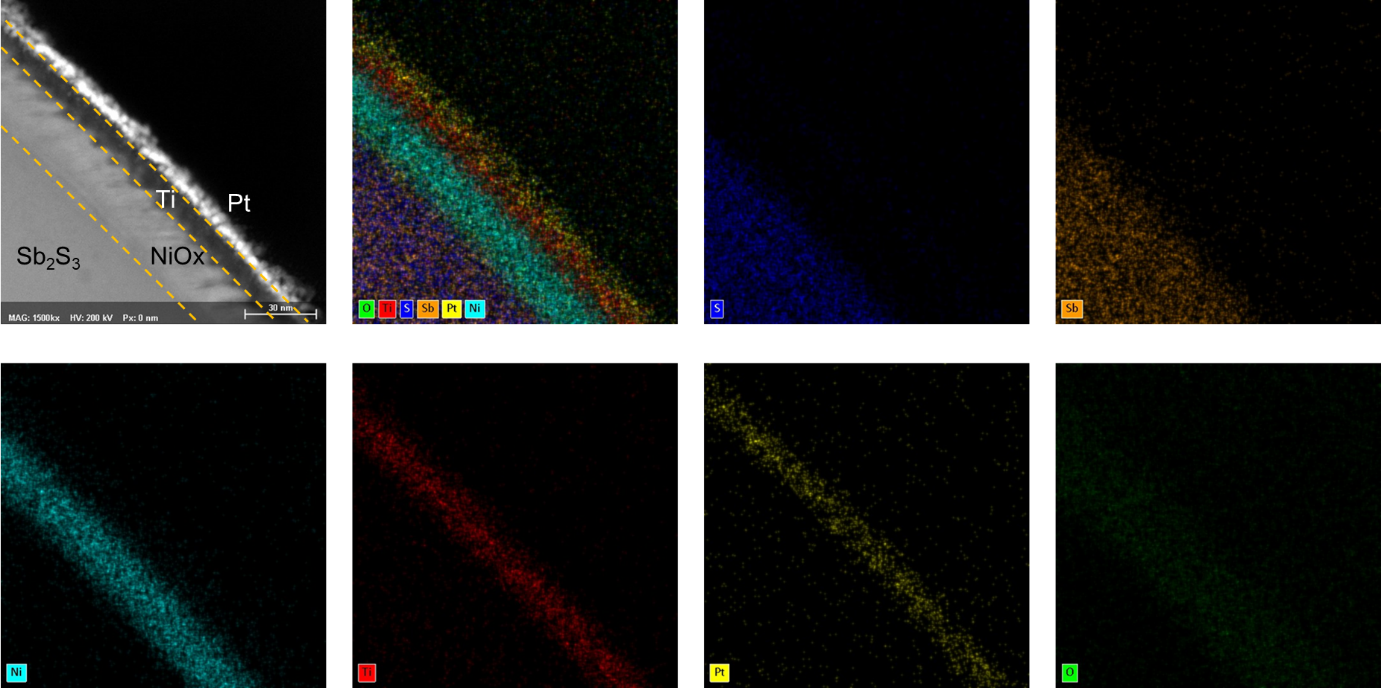


**Figure S12**. High-angle annular dark-field (HAADF) image and corresponding STEM-EDX mapping of the Sb_2_S_3_/NiOx/Ti/Pt interface showing elemental distribution.


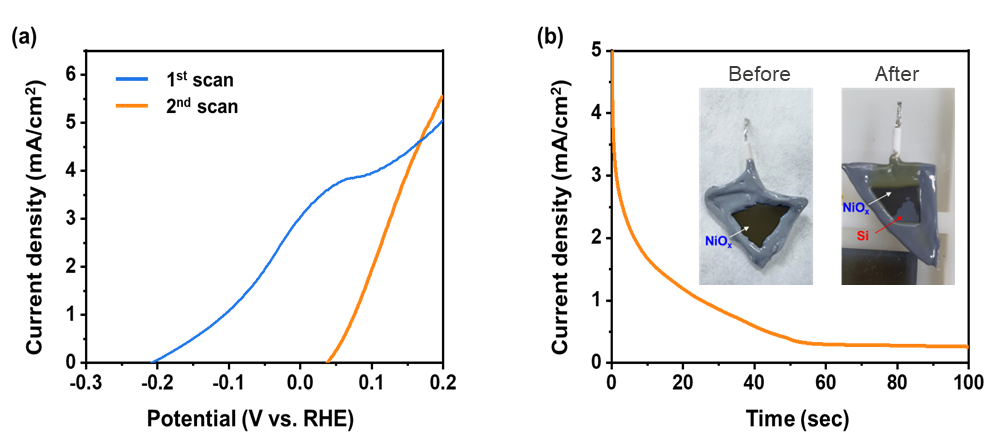


**Figure S13**. (a) LSV performance of Ag/ITO/Si/ITO/Au/Sb_2_S_3_/NiO_x_ tandem PEC device (without protection layer) for IOR reaction. (b) Short-term stability test under unbiased conditions.


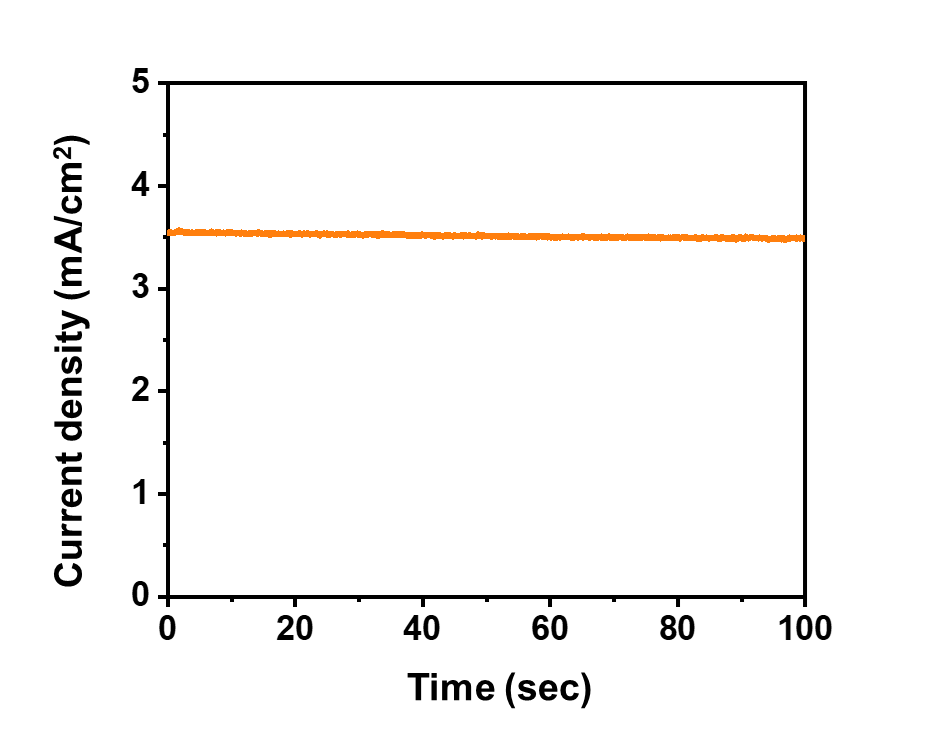


**Figure S14**. Short-term stability test of monolithic Ag/ITO/Si/ITO/Au/Sb_2_S_3_/NiO_x_/Ti/Pt tandem device with Ti protection layer under unbiased conditions.


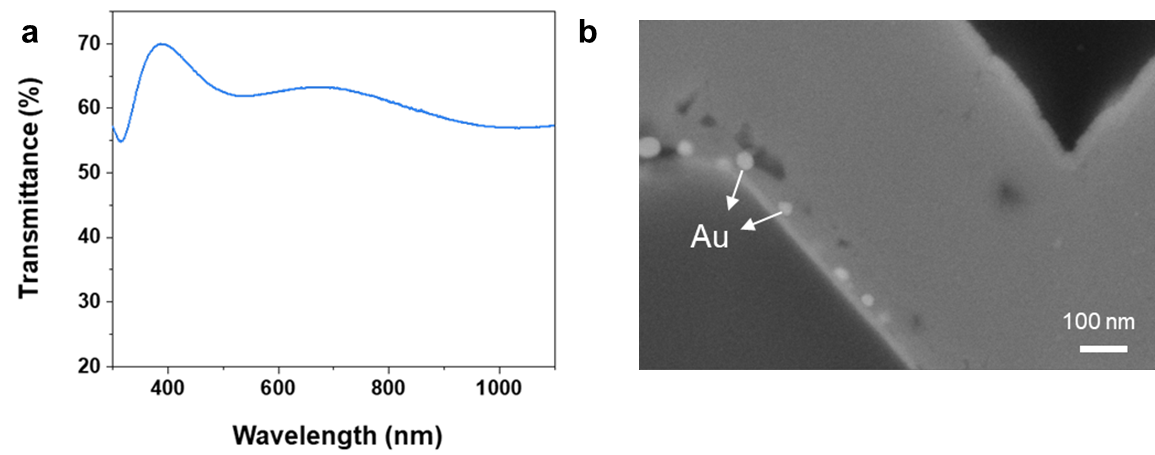


**Figure S15.** (a) UV–vis transmittance spectrum of the Ti (10 nm), Pt (2 nm) overlayer measured over 300–1100 nm. (b) 10 nm Au at the ITO recombination layer interface. The droplet-like morphology of Au layer does not pose optical limitations to the bottom silicon cell.





**Figure S16**. Performance of the tandem PEC device for coupled reactions without Au interlayer in the tandem stack (Ag/ITO/Si/ITO/Sb_2_S_3_/NiO_x_).


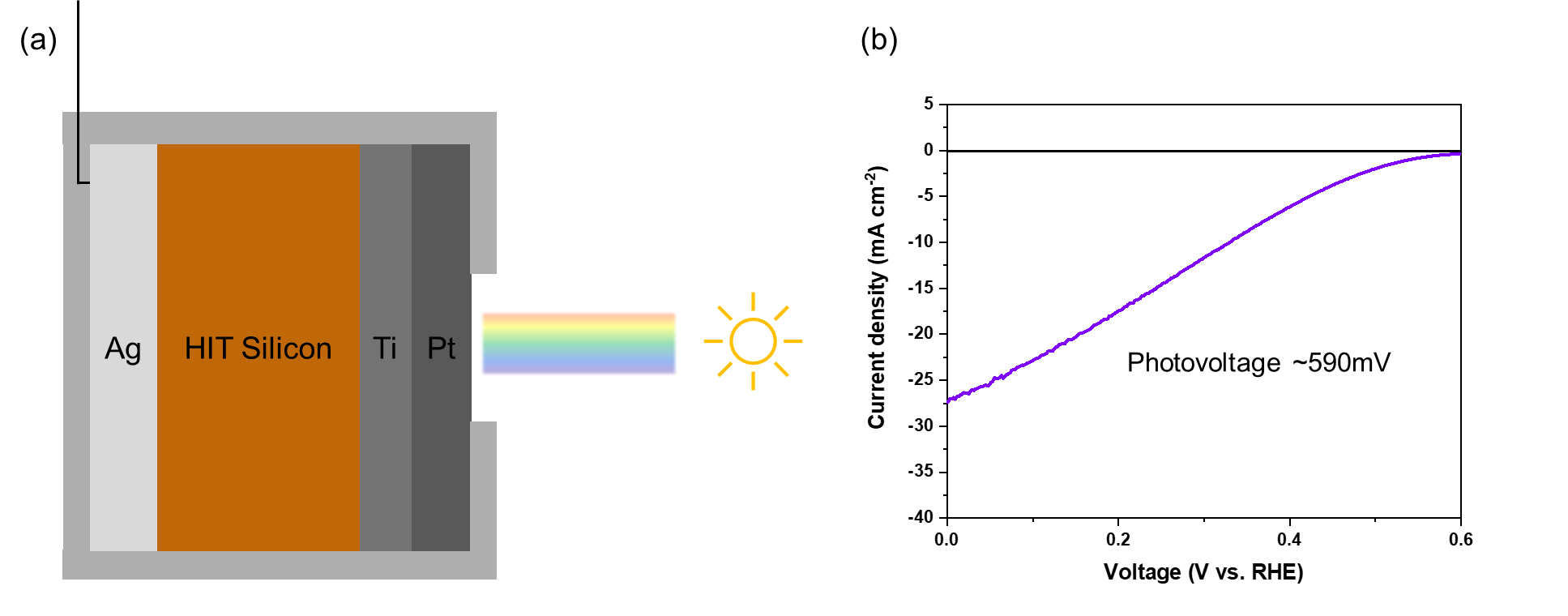


**Figure S17**. (a) Schematic illustration of the single photocathode fabricated from HIT silicon. (b) Linear sweep voltammetry (LSV) curve of the HIT silicon photocathode measured for the hydrogen evolution reaction (HER) in a three-electrode configuration in same electrolyte environment used for tandem IOR measurements.


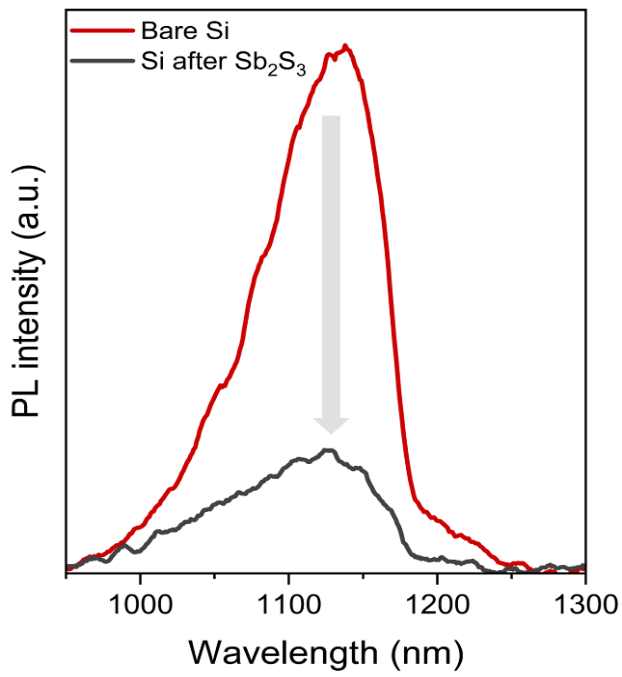


**Figure S18**. Photoluminescence spectra of the silicon sample before and after the Sb_2_S_3_ deposition.


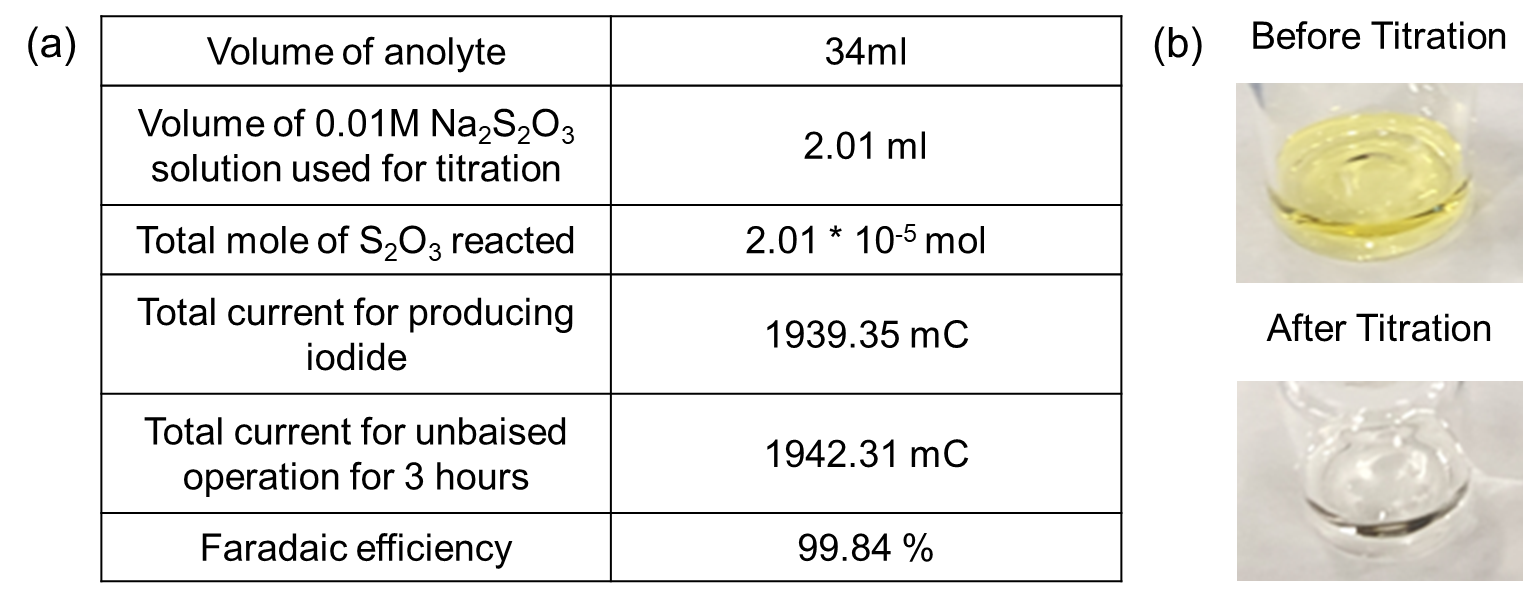


**Figure S19**. Iodometric titration analysis of iodine generated during unbiased PEC operation.

(a) Quantitative summary of the titration analysis after 3 h of bias-free PEC operation. After 3 h of bias-free PEC operation, the anolyte (34 mL) was collected and titrated with 0.01 M Na_2_S_2_O_3_ solution. A total of 2.01 mL of Na_2_S_2_O_3_ was required to reach the colorless endpoint from the initial yellow electrolyte, corresponding to $2.01\times{10}^{-5}$mol of reacted thiosulfate. Based on the reaction $I_{2}+2S_{2}O_{3}^{2-}\to2I^{-}+S_{4}O_{6}^{2-}$, this corresponds to 1939.35 mC of charge associated with iodine production. Compared with the total charge passed during 3 h of unbiased operation (1942.31 mC), the Faradaic efficiency was calculated to be 99.84%.

(b) Photographs of the anolyte before and after iodometric titration, showing the disappearance of the yellow color after titration with 0.01 M Na_2_S_2_O_3_, consistent with consumption of the oxidized iodine species.


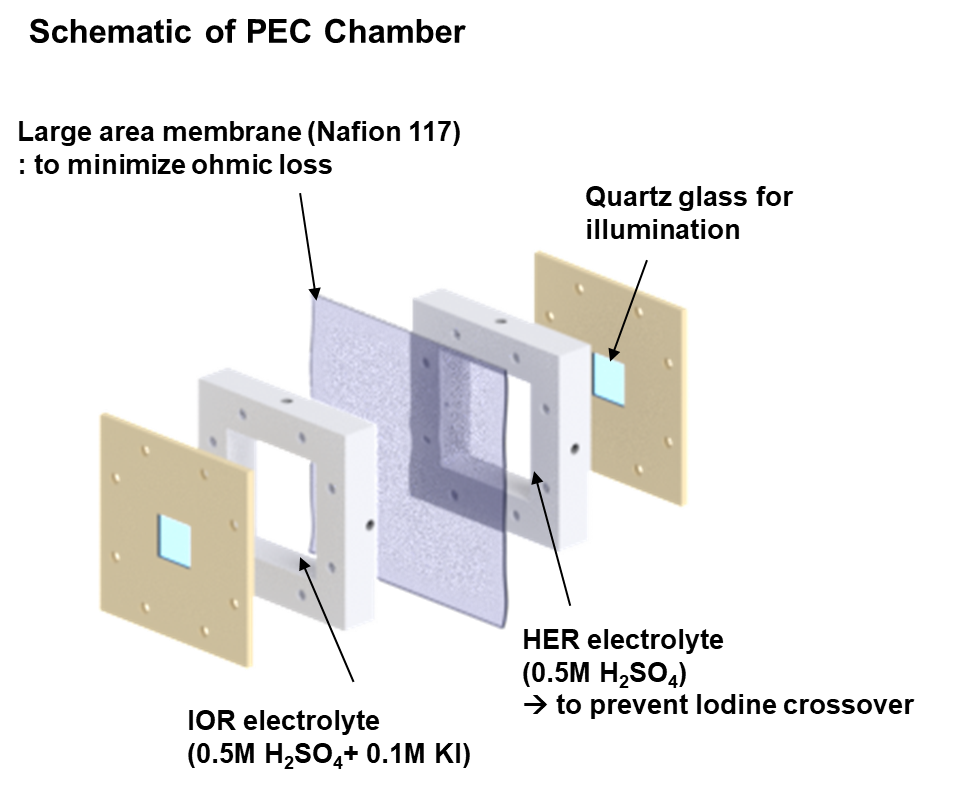


**Figure S20**. Detailed schematic illustration of the custom-designed PEC reactor chamber employed for unbiased solar-driven hydrogen and iodine co-production experiments. The chamber consists of two distinct compartments, separated by a large-area Nafion 117 membrane to effectively minimize ohmic losses and prevent crossover of reaction products between the anolyte and catholyte sides. The anolyte side (left compartment) contains iodide oxidation reaction (IOR) electrolyte composed of 0.5 M H_2_SO_4_ and 0.1 M KI, whereas the catholyte side (right compartment) is filled with hydrogen evolution reaction (HER) electrolyte consisting of 0.5 M H_2_SO_4_, specifically designed to prevent iodine crossover and undesired back reactions. A quartz glass window is integrated into the front side of the reactor to ensure high optical transparency, enabling efficient illumination of the photoelectrode during PEC measurements under simulated AM 1.5G (1 sun) conditions.


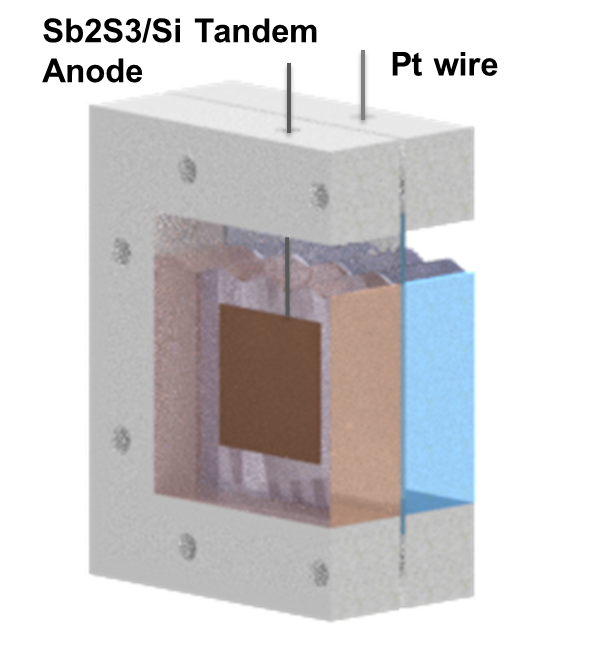


**Figure S21**. Detailed schematic representation of the two-electrode unbiased PEC measurement system designed for evaluating the solar-driven hydrogen and iodine co-production from the Sb_2_S_3_/Si tandem PEC device. The tandem PEC photoanode (Sb_2_S_3_/Si tandem electrode) is positioned within the anolyte compartment, while a platinum (Pt) wire is employed as the cathode in the catholyte compartment. The two compartments are separated by a membrane (Nafion 117), preventing undesirable product crossover and ensuring that only the desired hydrogen evolution reaction (HER) occurs at the cathode side. This configuration allows accurate assessment of the intrinsic performance and stability of the tandem PEC device under unbiased operating conditions.

References

1. Kresse, G.; Furthmüller, J. Efficient Iterative Schemes for Ab Initio Total-Energy Calculations Using a Plane-Wave Basis Set. *Phys. Rev. B* **1996**, *54* (16), 11169–11186. <https://doi.org/10.1103/PhysRevB.54.11169>.
2. Kresse, G. Ultrasoft Pseudopotentials to the Projector Augmented-Wave Method. *Phys. Rev. B* **1999**, *59* (3), 1758–1775. <https://doi.org/10.1103/PhysRevB.59.1758>.
3. Perdew, J. P.; Burke, K.; Ernzerhof, M. Quantum Theory Group Tulane University, N. O. L. 70118 J. Generalized Gradient Approximation Made Simple. *Phys. Rev. Lett.* **1996**, *77* (3), 3865–3868. <https://doi.org/10.1103/PhysRevLett.77.3865>.
4. Nitopi, S.; Bertheussen, E.; Scott, S.B.; Liu, X.; Engstfeld, A.K.; Horch, S.; Seger, B.; Stephens, I.E.L.; Chan, K.; Hahn, C.; Norskov, J.K.; Jaramillo, T.F. Progress and Perspectives of Electrochemical CO_2_ Reduction on Copper in Aqueous Electrolyte. *Chem. Rev.* **2019**, 119, 7610–7672. https://doi.org/10.1021/acs.chemrev.8b00705.
5. Gurudayal.; Beeman, J.W.; Bullock, J.; Wang, H.; Eichorn, J.; Towle, C.; Javey, A.; Toma, F.M.; Mathews, N.; Ager, J.W. Si photocathode with Ag-supported dendritic Cu catalyst for CO_2_ reduction. *Energy Environ. Sci.* **2019**, 12, 1068–1077. https://doi.org/10.1039/C8EE03547D.
